# CRISPR/Cas9‐mediated tetra‐allelic mutation of the ‘Green Revolution’ *SEMIDWARF‐1* (*SD‐1*) gene confers lodging resistance in tef (*Eragrostis tef*)

**DOI:** 10.1111/pbi.13842

**Published:** 2022-06-10

**Authors:** Getu Beyene, Raj Deepika Chauhan, Justin Villmer, Nada Husic, Ning Wang, Endale Gebre, Dejene Girma, Solomon Chanyalew, Kebebew Assefa, Girma Tabor, Malia Gehan, Michael McGrone, Meizhu Yang, Brian Lenderts, Chris Schwartz, Huirong Gao, William Gordon‐Kamm, Nigel J. Taylor, Donald J. MacKenzie

**Affiliations:** ^1^ 7538 Donald Danforth Plant Science Center St. Louis MO USA; ^2^ 57705 Corteva Agriscience Johnston IA USA; ^3^ 3078 Michigan State University East Lansing MI USA; ^4^ 128161 Ethiopian Institute of Agricultural Research Addis Ababa Ethiopia

**Keywords:** *Eragrostis tef*, lodging, *SEMIDWARF‐1*, CRISPR/Cas9, *BABY BOOM*, *WUSCHEL*

## Abstract

Tef is a staple food and a valuable cash crop for millions of people in Ethiopia. Lodging is a major limitation to tef production, and for decades, the development of lodging resistant varieties proved difficult with conventional breeding approaches. We used CRISPR/Cas9 to introduce knockout mutations in the tef orthologue of the rice *SEMIDWARF‐1* (*SD‐1*) gene to confer semidwarfism and ultimately lodging resistance. High frequency recovery of transgenic and *SD‐1* edited tef lines was achieved in two tef cultivars by *Agrobacterium*‐mediated delivery into young leaf explants of gene editing reagents along with transformation and regeneration enhancing morphogenic genes, *BABY BOOM* (*BBM*) and *WUSCHEL2* (*WUS2*). All of the 23 lines analyzed by next‐generation sequencing had at least two or more alleles of *SD‐1* mutated. Of these, 83% had tetra‐allelic frameshift mutations in the *SD‐1* gene in primary tef regenerants, which were inherited in subsequent generations. Phenotypic data generated on T_1_ and T_2_ generations revealed that the *sd‐1* lines have reduced culm and internode lengths with no reduction in either panicle or peduncle lengths. These characteristics are comparable with rice *sd‐1* plants. Measurements of lodging, in greenhouse‐grown plants, showed that *sd‐1* lines have significantly higher resistance to lodging at the heading stage compared with the controls. This is the first demonstration of the feasibility of high frequency genetic transformation and CRISPR/Cas9‐mediated genome editing in this highly valuable but neglected crop. The findings reported here highlight the potential of genome editing for the improvement of lodging resistance and other important traits in tef.

## Introduction

Tef [*Eragrostis tef* (Zucc.), Trotter] is an ancient grain cultivated in Ethiopia as a major staple food and cash crop for millions and a valued forage crop (Demeke and Marcantonio, 2019; Minten *et al*., 2018). Tef ranks first among cereals in the area of production in Ethiopia, covering about three million hectares and cultivated by more than six million farmers (Minten *et al*., 2018). Tef is tolerant to pests and diseases and suffers minimal post‐harvest losses (Assefa and Chanyalew, 2018). Tef can grow relatively well in extremes of conditions not suitable for other cereals including a wider range of altitudes (up to 3000 m above sea level) and moisture extremes (Assefa *et al*., 2015), consequently rendering this grass more climate resilient than most crops. Tef grain is also valued as a high protein source, with a balanced and complete set of amino acids, and is a rich source of minerals including iron and calcium and is gluten‐free, which contributes to food products suitable for people with celiac disease (Baye, 2014).

Over 50 years of research and development between 1960–2012 have brought incremental improvements in tef grain yield with genetic gains of 0.5 to 0.79 percent per year (Assefa and Chanyalew, 2018; Teklu and Tefera, 2005). However, tef yield remains very low compared to that of the other major cereals, averaging about 1.7 t/ha (Assefa *et al*., 2011; Cochrane and Bekele, 2018). Lodging, competition from weeds, grain shattering and low productivity of landraces (the types predominantly cultivated by farmers) are major limitations to tef production (Assefa *et al*., 2011). Lodging suppresses yield on average by 17% and substantially reduces the quality of harvested grain and straw (Assefa *et al*., 2011; Ketema, 1993).

Available tef germplasm pool manifests considerable genetic diversity in plant height, days to maturity, panicle type, caryopsis color and seed size, and plant characteristics contributing to lodging resistance/susceptibility (Assefa *et al*., 1999, 2011, 2015; Bayable *et al*., 2020). Decades of conventional breeding approaches to improve lodging resistance in tef have been of limited success (Assefa *et al*., 2011; Ketema, 1991; Tefera *et al*., 2003; Zhu *et al*., 2012). Recently, chemical mutagenesis has resulted in the successful development of a lodging resistant dwarf tef variety defective in the *α‐tubulin* gene (Jöst *et al*., 2015). Introgression of this mutation into elite tef germplasm and agronomic evaluation is underway (Bekana and Assefa, 2021). Currently, all improved varieties of tef lodge to a varying degree (Cochrane and Bekele, 2018).

To combat lodging susceptibility, agronomically useful dwarfing genes have been extensively used in cereal crops (Liu *et al*., 2018; Quinby and Karper, 1954). The sources of the dwarfing genes are mainly spontaneous mutations, some of which have been under cultivation for about a century (Yamaguchi *et al*., 2016), while some were selected by humans thousands of years ago during early crop domestication (Asano *et al*., 2011). Semidwarf lines of different crop species have also been developed through induced mutagenesis (Shu *et al*., 2012). The most widely deployed and well‐studied dwarfing genes are the *reduced height‐1* (*Rht‐1*) gene in wheat (Peng *et al*., 1999) and *sd‐1* gene in rice (Ashikari *et al*., 2002; Sasaki *et al*., 2002; Spielmeyer *et al*., 2002) both of which were central to the Green Revolution of the 1960s and 1970s (Hedden, 2003). The wild‐type alleles of these genes are involved in gibberellin (GA) signalling (*RHT‐1,* a DELLA protein) and biosynthesis (*SD‐1,* a GA 20‐oxidase). Likewise, the semidwarfing gene *uzu*, a brassinosteroid (BR)‐*insensitive1* (*BRI1*) gene encoding a BR receptor (Chono *et al*., 2003), and *sdw1*/*denso*, a GA 20‐oxidase orthologous to rice *sd‐1* (Jia *et al*., 2015), have both been used in barley breeding. On the contrary, mutations in GA biosynthetic genes causing GA deficiency were not useful in sorghum, due to associated undesirable stem growth phenotype (Ordonio *et al*., 2014). Rather, four independently inherited non‐GA dwarfing genes (*DW1–DW4*), have been used extensively in commercial grain sorghum breeding mainly in the USA to significantly reduce sorghum plant height to improve lodging resistance and machine harvesting (Quinby and Karper, 1954). Cloning of these genes revealed that *DW3* is an ortholog to maize *BRACHYTIC2 (BR2)*, which encodes for ABCB1 auxin transporter (Multani *et al*., 2003), that *DW1* encodes for a novel component of BR signalling (Hirano *et al*., 2017a) and *DW2* encodes for AGC protein kinase involved in the regulation of stem elongation (Hilley *et al*., 2017). The gene encoding DW4 has not been cloned and its identity remains unknown. Studies of dwarfing genes have shown that *sd‐1*, *dw1*, *dw2* and *dw3* are recessive, making them good candidates for targeted knockout mutagenesis using genome editing technologies. In tef, application of GA biosynthesis inhibitors, such as chlormequat chloride and paclobutrazol, have generated semidwarf and lodging resistant tef plants (Gebre *et al*., 2012; Plaza‐Wüthrich *et al*., 2016), indicating that gibberellin deficiency induced through mutations in the tef *SD‐1* orthologue could be an avenue for generating semidwarf, lodging resistant tef lines.

Recent advances in genome engineering technologies, including CRISPR/Cas9 (clustered regularly interspaced short palindromic repeats/CRISPR associated‐9) and transcription activator‐like effector nucleases (TALENS), have revolutionized the field of basic and applied plant biology. Plant traits of significance have been improved through this technology (Zhu *et al*., 2020). These, include resistance to bacterial blight in rice (Oliva *et al*., 2019), resistance to powdery mildew in wheat (Wang *et al*., 2014), virus resistance in cassava (Gomez *et al*., 2019), improvements in food quality traits in potato and soybean (Clasen *et al*., 2016; Haun *et al*., 2014), improvements in maize starch profile (Gao *et al*., 2020) and agronomic performance (Shi *et al*., 2017) including yield and yield components (Liu *et al*., 2021). Rapid domestication of underutilized crops has also been reported, demonstrating the potential of genome editing to improve the livelihoods of low‐income farmers (Østerberg *et al*., 2017; Zsögön *et al*., 2018).

We report here the first successful production of semidwarf tef lines using CRISPR/Cas9 based genome editing of tef *SD‐1* gene. We present data on the nature and heritability of the mutations obtained and demonstrate the promise of genome editing for conferring lodging resistance in tef.

## Results

### Tef GA 20‐oxidases

**Figure 1:**
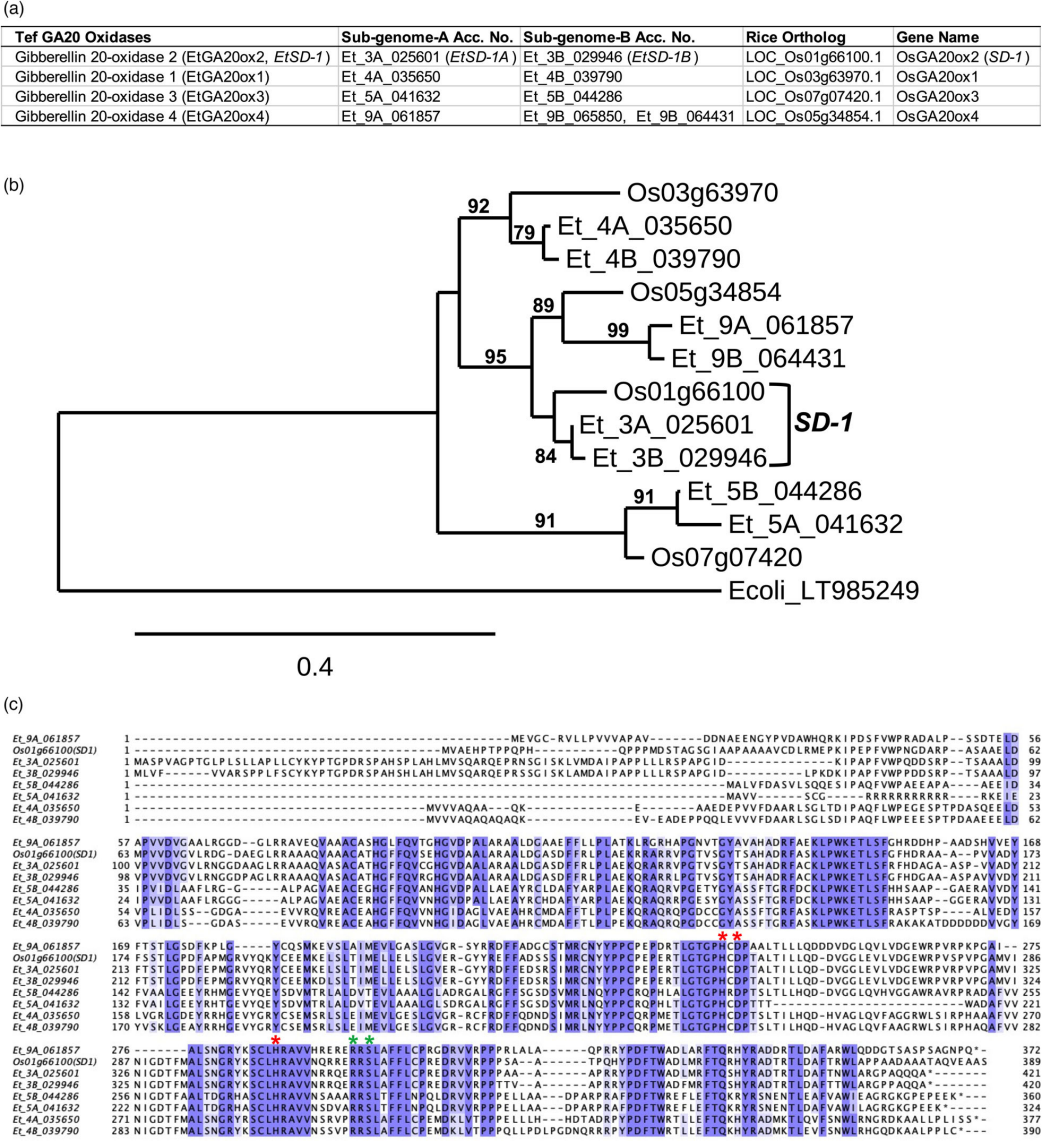
Tef and rice GA 20‐oxidases. The four tef GA 20‐oxidases (*EtGA20ox1‐4*) and corresponding rice orthologs (a), phylogenetic tree showing grouping of each of the tef GA 20‐oxidases with corresponding rice orthologs (b). Note, rice *SD‐1* grouped with *EtGA20ox2*. Multiple sequence alignment of tef GA 20‐oxidases along with rice *SD‐1* showing conservation of critical amino acid residues (c). Multiple sequence alignment was run using Clustal Omega (Madeira *et al*., 2019), and aligned sequence was edited in sequence alignment viewer Jalview (Waterhouse *et al*., 2009). The highly conserved HX(D/E)X_n_H triad motif (marked with red star), essential for binding Fe(II) and the two residues (marked with green star) involved in 2‐oxoglutarate binding (c) common to other 2‐oxoglutarate/Fe(II)‐dependent dioxygenases (2‐ODDs) described by Farrow and Facchini (2014) are shown.

Using the rice GA 20‐oxidases (*GA20ox1‐4*, Sakamoto *et al*., 2004a, 2004b) coding sequences (CDS) as baits, four tef GA 20‐oxidases were identified from the tef genome (VanBuren *et al*., 2020) using CoGeBlast (Lyons *et al*., 2008). Based on nucleotide and deduced amino acid sequence identities, these GA 20‐oxidases were named *EtGA20ox1‐4*, with the corresponding rice GA 20‐oxidases as shown in Figure 1a. Designation of the homeologs of each of the four tef GA 20‐oxidases followed the corresponding A or B genomes. The four tef GA 20‐oxidases CDS shared 61.7%–75.3% (Table S1) and 46.5%–69.3% amino acid identity with each other (Table S2). The level of similarity between each of the two tef homoeologous alleles (*EtGA20ox1*, *EtGA20ox2* and *EtGA20ox3*) CDS ranged from 90.8%–95.4% nucleotide sequence identity and 88.2%–92.3% amino acid sequence identity (Tables S1 and S2). Based on the reference genome sequences, *EtGA20ox4* has only one functional allele (Et_9A_061857), with the second allele represented by two shorter open reading frames (ORFs), Et_9B_065850 and Et_9B_064431 (Figure 1a) due to a mutation introducing a premature stop codon. The wild‐type rice *SD‐1* (*OsGA20ox2*) was found to be closely related to *EtGA20ox2* (*EtSD‐1,* Figure 1a,b) with 83.5% nucleotide and 81.7%–84.4% amino acid identity, compared to that of the other tef GA 20‐oxidases that shared 66%‐76.3% nucleotide and 48.6%–66% amino acid identities (Tables S1 and S2).

The tef *SD‐1* (*EtSD‐1*) was chosen as a target for knockout mutation based on its similarity with the rice *SD‐1* gene. All identified tef GA 20‐oxidases (*EtGA20ox1‐4*) had the highly conserved HX(D/E)X_n_H triad motif, essential for binding Fe(II) and the two residues involved in 2‐oxoglutarate binding (Figure 1c) common to 2‐oxoglutarate/Fe(II)‐dependent dioxygenases (2‐ODDs; Farrow and Facchini, 2014).

### Recovery of SD‐1 edited tef lines

**Figure 2:**
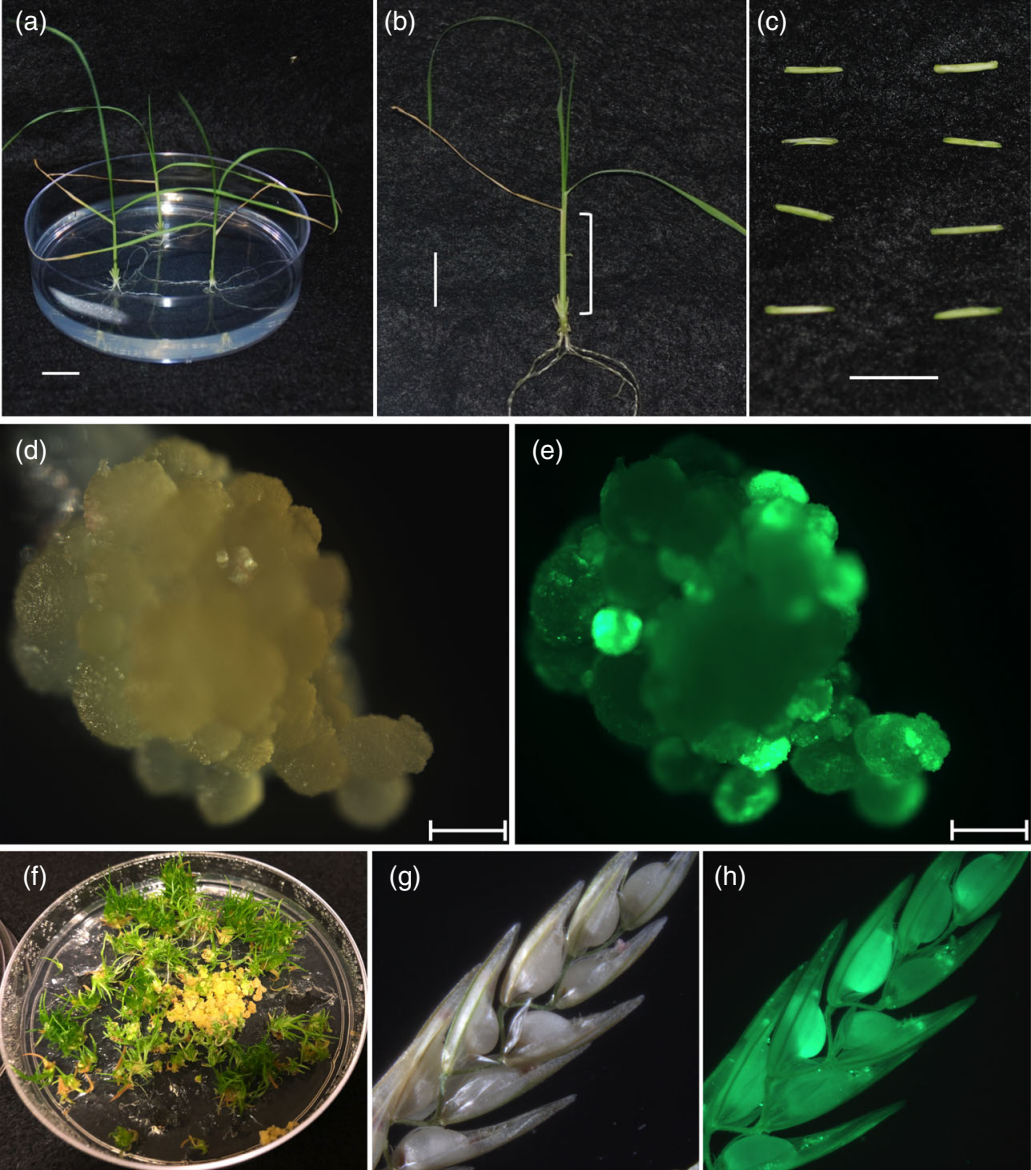
Tef plant transformation and recovery of transgenic events. Two‐ to three‐week‐old tef seedlings grown aseptically (a) portion of young leaves (folded young leaves or stems) used as a source of explants (b) excised and longitudinally sectioned young leaf explants ready for *Agrobacterium*‐mediated transformation (c), white light and UV‐light images of sectors of callus expressing green fluorescent protein (GFP) after 4–5 weeks culture on callus induction media (d, e), regenerating tef plantlets developing on regeneration media (f) and white light (g) and UV‐light (h) pictures of transgenic tef panicle showing T_1_ seeds segregating for GFP expression obtained from 12‐week‐old T_0_ plants grown in the growth chamber. Scale bars in a, b and c = 1 cm, in d and e = 1 mm.

A simple *Agrobacterium*‐mediated tef transformation and regeneration system were developed using young leaf tissue as explants (Figure 2) excised from 2‐ to 3‐week‐old seedlings (Figure 2a). The leaf base portion of the seedlings (Figure 2b) was sectioned transversely into 4–8 mm long segments, split into halves (Figure 2c) and inoculated with *Agrobacterium* harbouring either p8660 or p8702. Both binary vectors had the same Cas9 and gRNAs but differed in the promoters used to drive the morphogenic genes *BBM*, *WUS2* and the *Cre*‐recombinase (Figures S1 and S2). After transformation, green fluorescent protein (GFP) foci were visible within 3–5 days from both constructs. The GFP expressing cells grew into sectors of rapidly dividing embryogenic tissue (Figure 2d,e). Since negative selection was not used in this study (no antibiotic or herbicide selection), the rapidly growing GFP expressing sectors were excised manually under a fluorescence microscope from nontransformed tissues and subcultured onto fresh callus induction medium or regeneration medium. Transgenic GFP expressing tissues were regenerated (Figure 2f) after induction of *Cre*‐recombinase expression to facilitate the excision of morphogenic genes. Rooted transgenic T_0_ plants were established in the soil, grown to flower stage and T_1_ seeds collected, which were shown to segregate for the GFP marker (Figure 2g,h).

On average, it took 6–8 months to complete the entire process from wild‐type seed germination through transformation and regeneration to the production of T_1_ transgenic seeds. Using this method, a total of 112 GFP expressing T_0_ tef plant lines were recovered in two tef cultivars (41 in Ada and 71 in Magna) with an average transformation efficiency using construct p8702 of 7.8% for Ada and 16.5% for Magna (Table S3). Of these, 20 T_0_ lines generated in Magna cultivar and seven lines in Ada cultivar were analyzed for mutations at the gRNA1 and gRNA2 target sites and characterized through subsequent generations. All the 27 lines were fertile and produced seeds, and four lines derived from p8660 in Magna background had delayed flowering (heading time), stunted growth, dark green leaves and produced less seeds than the wild‐type control. These lines were excluded from further phenotyping studies.

### High‐efficiency tetra‐allelic mutation in the Tef SD‐1 gene

**Figure 3:**
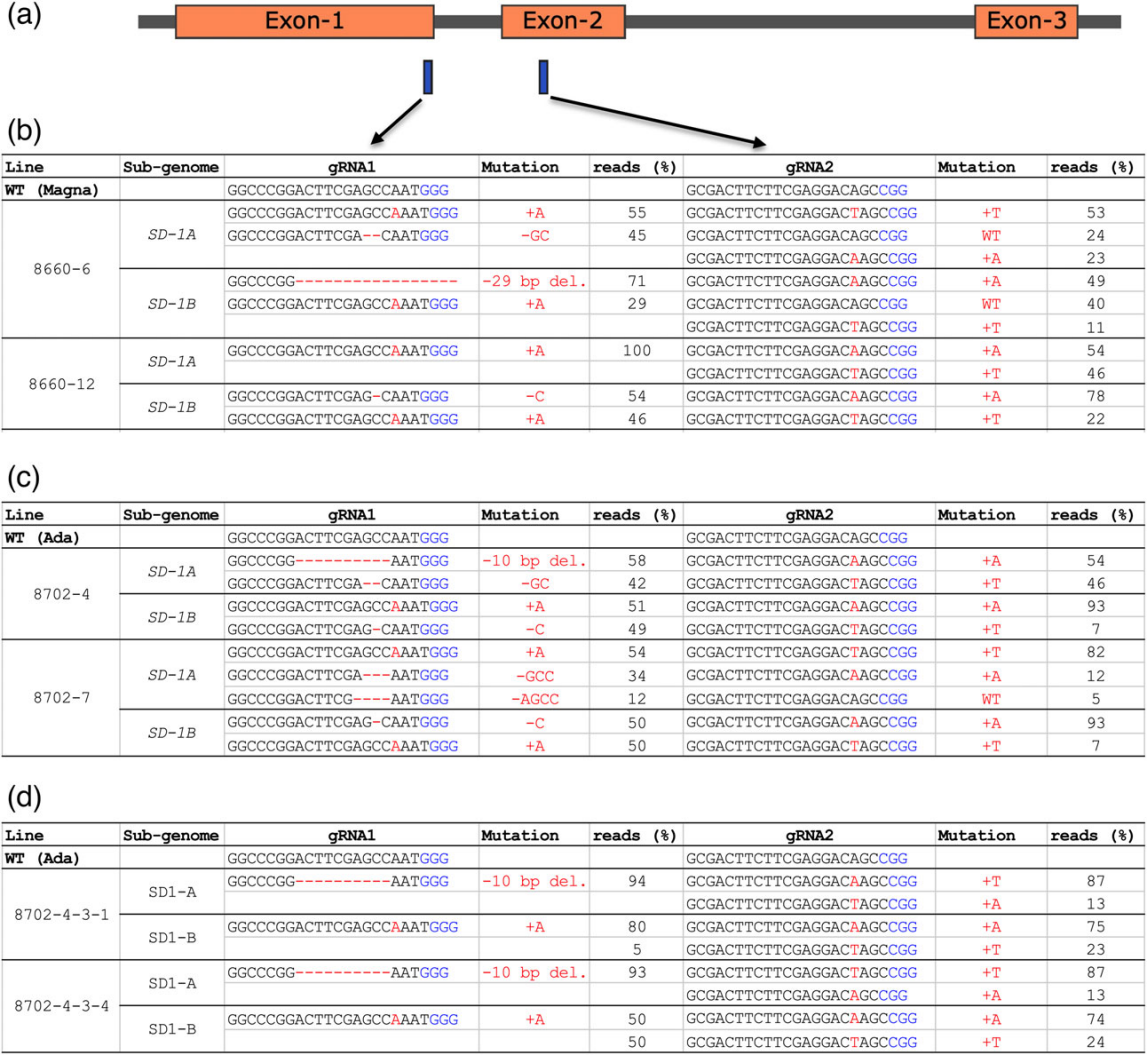
Mutations in the tef *SD‐1* gene at two target sites (gRNA1 and gRNA2). Schematic presentation of tef *SD‐1* showing relative positions of gRNA1 and gRNA2 (a), on exon‐1 and exon‐2, respectively; insertion (+) deletion (−) generated at the two target sites (red font) in T_0_ Magna edited plants (b) T_0_ Ada edited plants (c) T_2_ Ada edited plants (d). Read percent of each of the mutation calls were determined from next‐generation sequencing (NGS) data generated at each respective generation for the target site. Nucleotides in blue are the protospacer adjacent motive (PAM) site. Note chimera seen at gRNA2 site.

Two gRNAs were used to generate knockout mutations in the tef *SD‐1* gene. Positions of the *SD‐1* target site corresponding to exon‐1 (gRNA1 target site) and exon‐2 (gRNA2 target site) are shown in Figure 3a. Next‐generation sequencing of the two target site amplicons from both subgenomes, A and B, revealed unprecedented editing efficiency of 100% as all lines had at least two or more alleles mutated at the target site. Characterization of the mutations across the two cultivars showed 19 of the 23 lines (83%) had tetra‐allelic frameshift mutations (Figure 3b,c and Table S4). We also found four of the 27 lines (two in Ada and two in Magna) could potentially be clones based on next‐generation sequence data. The frequency of tetra‐allelic mutation was generally higher at the gRNA1 target site than at gRNA2 with 83% versus 56%, respectively. In almost all of *SD‐1* edited tef lines, the mutation types were simple insertion or deletions (InDels, Figure 3b,c, and Table S4). Insertions were mainly of single nucleotides while deletions ranged from 1 to 32 bp (Figure 3b,c, and Table S4). Chimeric mutations were also observed in some of the lines, mainly at the gRNA2 site. The observed mutations were heritable as confirmed through sequencing of subsequent generations (Figure 3d).

### SD‐1 mutation reduced culm length and plant height

**Figure 4:**
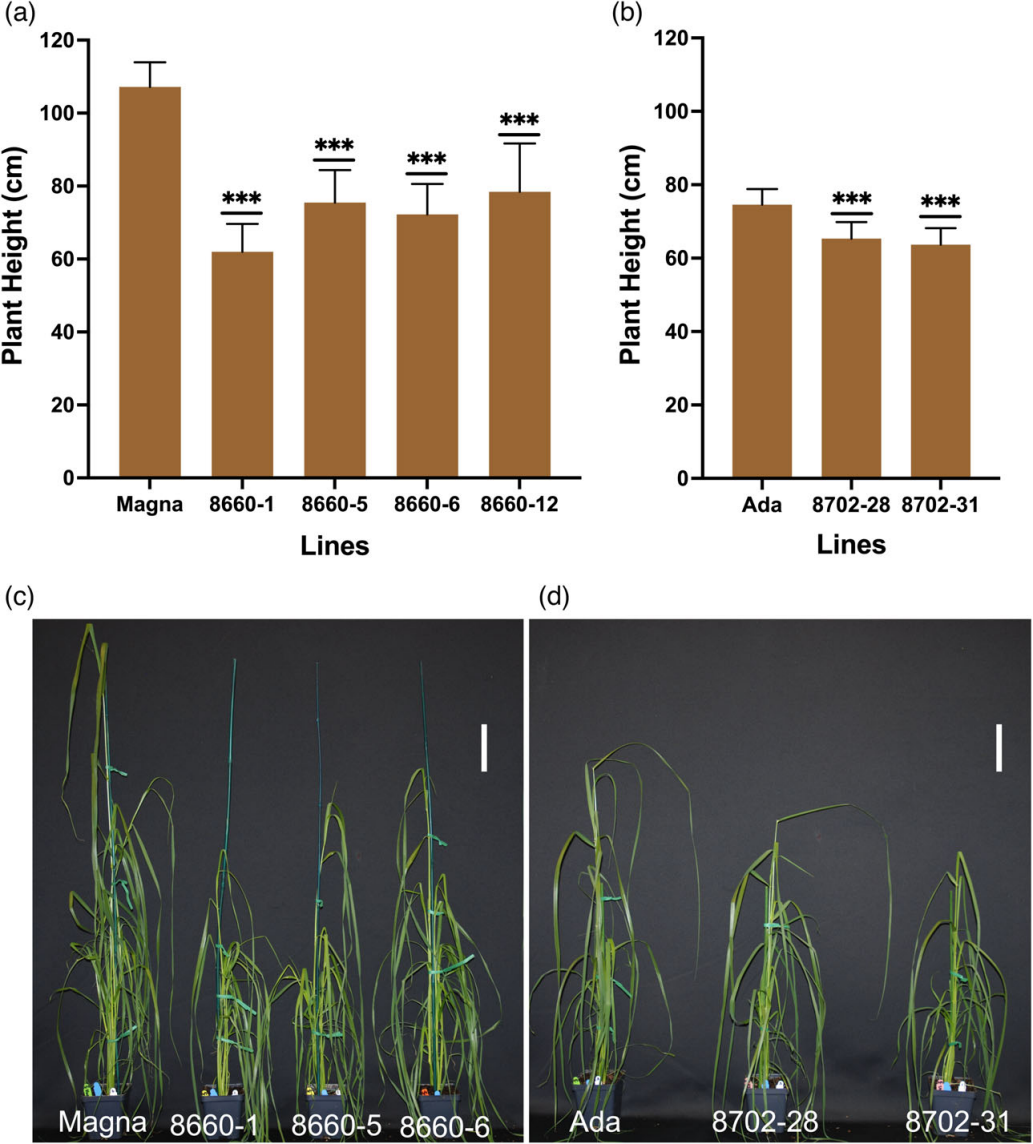
Tef plant height at vegetative growth stage in T_1_ generation plants after 5 weeks of growth in the growth chamber. Performance of tetra‐allelic *sd‐1* mutated lines generated from Magna (a, c) and Ada (b, d) with respective wild‐type controls. Plant height was measured from stem base at soil level to the topmost collar of the main stem. Bars show mean ± SD, *n = *18–36. ***, denotes for significant differences compared with the wild‐type cultivar at *P* < 0.001. Scale bars in (c) and (d) = 10 cm.

Null mutation in the *SD‐1* gene is known to affect culm and internode lengths in rice (Tomita and Ishii, 2018). Phenotypic effects of the *SD‐1* mutations in tef were evaluated by measuring four T_1_ Magna and two Ada lines for stem height during vegetative growth, and for culm, internode and panicle lengths at maturity (harvest). These lines had tetra‐allelic knockouts at T_0_ and were subsequently confirmed through sequencing to carry the *SD‐1* knockout in the T_1_ progenies (Figure 3b,c and Table S4). There were no differences in germination rates between T_1_ seed from edited *sd‐1* lines and wild‐type seed (data not shown). Visible differences in plant height started to emerge at about 2–3 weeks after planting. Measurements of stem height (as measured from soil level to the topmost collar) at 37 days after planting revealed that *sd‐1* lines in Magna background had a significant (*P* < 0.001) reduction in plant height (28%–42%; Figure 4a,c). In addition, two *sd‐1* Ada lines, 8702‐28 and 8702‐31, had 12% and 15% reductions (*P* < 0.001) in plant height compared to that in the unmodified Ada control (Figure 4b,d). Further characterization on T_2_ lines was conducted on Magna edited lines only as those derived from Ada lines (both edited and wild‐type) suffered poor panicle exertion under the conditions of our study.

**Figure 5:**
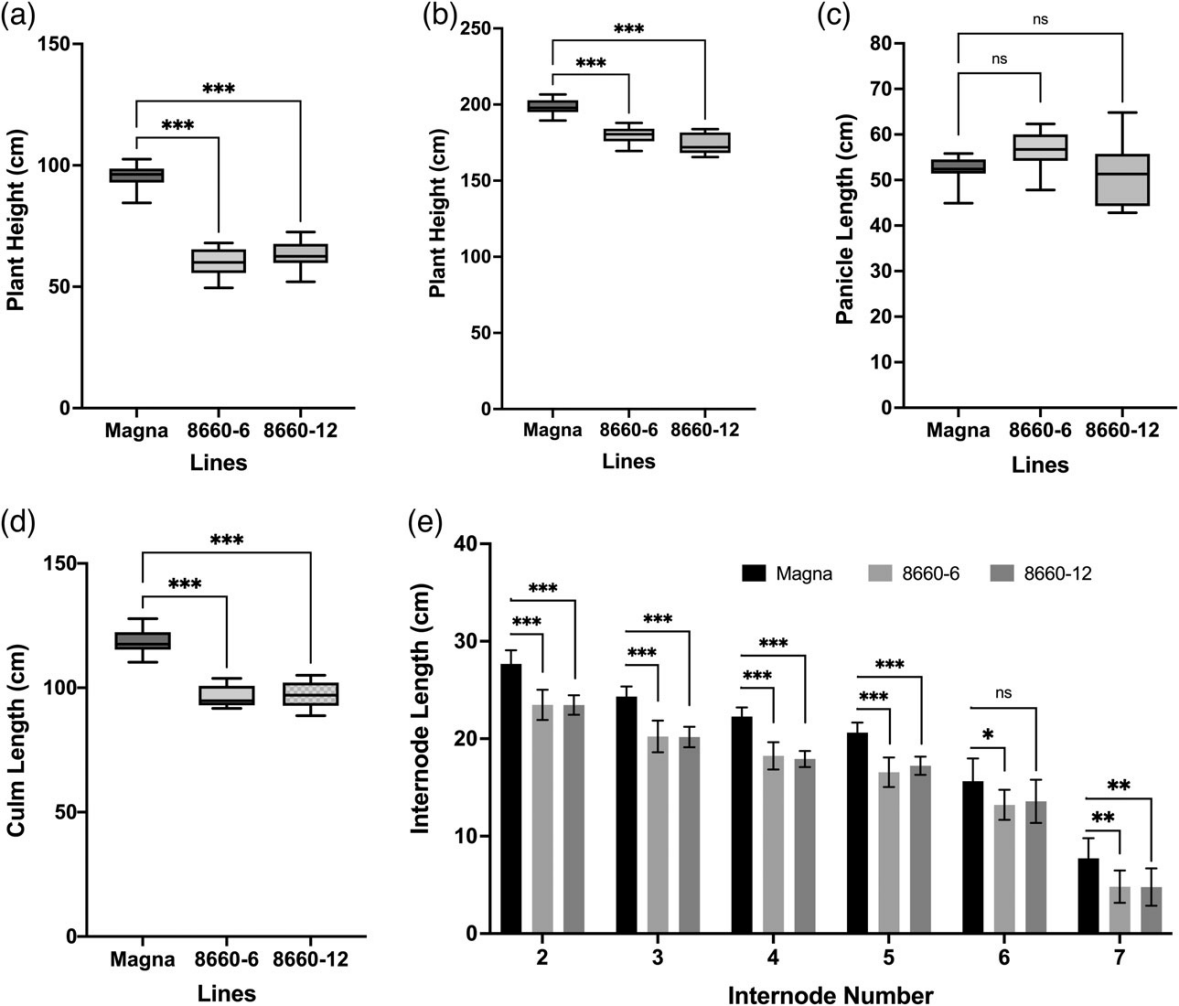
Tef plant height and internode, culm and panicle lengths in T_2_ tetra‐allelic *sd‐1* lines and wild‐type Magna. Plant height at 5 weeks after planting (a), plant height (b) panicle length (c), culm length (d) and lengths of each of the internodes from internode number 2 through 7 (e) measured at harvest 13 weeks after planting from staked plants. Internode length is the length of internodes between two consecutive nodes. Culm length is the sum of the lengths of all internodes without internode #1 (peduncle) and panicle lengths. Plant height was recorded by adding the sum of all internodes including peduncle and panicle lengths. Measurements were taken on 10 plants per line, three stems per plant (pot). *, ** and ***, indicate significant differences compared with the wild‐type Magna at *P* < 0.05, *P* < 0.01, *P* < 0.001. ns, stands for nonsignificant differences at *P* < 0.05.

Quantitative PCR analysis identified null segregants in Magna *sd‐1* T_1_ generation lines. Of these, two null segregant T_2_ lines, 8660‐6 and 8660‐12 were subjected to further phenotypic characterization. Like the T_1_ generation, discernable plant height differences were observed in the T_2_ generation at the vegetative growth stage 2–3 weeks after planting. Measurements of plant height at 5 weeks showed significant (*P* < 0.001) differences between the wild‐type and *sd‐1* lines, with 37% and 33% reductions in height relative to the Magna control for lines 8660‐6 and 8660‐12, respectively (Figure 5a). At harvest, the *sd‐1* lines had 10%–12% reduction in plant height (Figure 5b), 18% reduction in culm length (Figure 5d), and no significant difference in panicle (Figure 5c) and in peduncle lengths (data not shown) compared to that in the Magna control. Since culm length is the sum of all internodes (internodes 2–7) without the peduncle, we examined whether all, or only some, of the internodes were reduced in length. Individual internode lengths were not significantly different between the two *sd‐1* edited lines but were significantly reduced compared to that of the Magna control (Figure 5e). Average percent reductions in the lengths of internodes 2 through 6 ranged between 14%–19%, with no correlation between internode number and percent reduction in internode length. The average percent length reduction of basal internode‐7 was seen to be significantly greater at 38%. Under growth chamber conditions, tiller number (both total and effective tillers) was not significantly different between *sd‐1* lines and the Magna control (data not shown).

**Figure 6:**
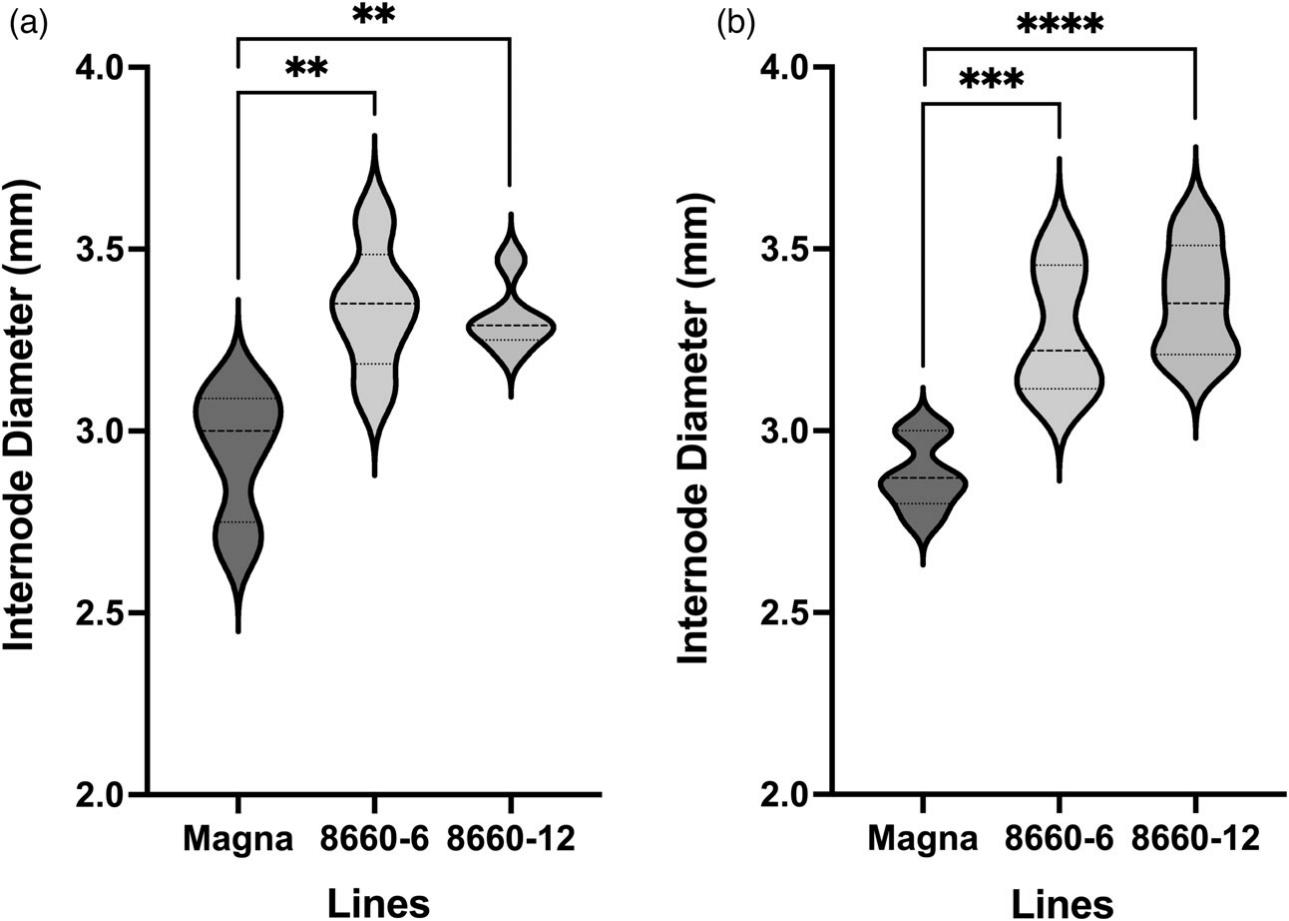
Internode diameter of *sd‐1* tef lines and Magna control. Internode diameter of internode number 9 (first internode above the soil) (a) and Internode 8 (second internode above the soil) (b) of *sd‐1* tef lines and the wild‐type Magna control. Data were collected using caliper for external diameter. Bars are SD, *n* = 5–7. **, *** and **** stand for significant differences, respectively, at *P* ≤ 0.01, *P* ≤ 0.001 and *P* ≤ 0.0001. The Student's *t*‐test was used for comparison.

Internode diameter at harvest was measured on the *sd‐1* edited lines and controls from the two consecutive internodes at the base of greenhouse‐grown T_2_ plants (internode‐8 or ‐9). The *sd‐1* lines had significantly (*P* < 0.01) thicker internodes compared to that of the corresponding internodes of the wild‐type Magna control (Figure 6a,b). No differences were observed in internode thicknesses at the mid‐section of the plant (internode‐3; data not shown). It was noted that both *sd‐1* lines and the wild‐type control had a greater number of nodes (8 or 9) under greenhouse conditions than observed from plants grown in the growth chamber (up to seven nodes).

### Application of GA_3_ rescued semidwarf phenotype of sd‐1 lines

To study whether the semidwarfism seen in *sd‐1* lines could be rescued by application of GA_3_, two experiments were run. In the first experiment, GA_3_ was applied as a spray treatment for 11 consecutive days to greenhouse‐grown 6‐week‐old wild‐type Magna and the semidwarf line 8660‐6 and increases in stem length were recorded. In GA_3_‐untreated control (−GA_3_) stem length (measured as a difference in plant height before and after treatment) was increased by 34.2 cm for 8660‐6 and by 55.5 cm in wild‐type Magna. In GA_3_‐treated plants (+GA3) of 8660‐6 and wild‐type Magna, stem length was increased by 87.5 and 95.9 cm, respectively. Relative to untreated controls, GA_3_ treatment increased stem elongation more in line 8660‐6 (155% increase) than in wild‐type control (82% increase), offsetting the effect of *SD‐1* knockout on stem elongation (Figure S3a,b). In the second experiment, *in vitro* germinated seedlings (from T_4_ generation) of *sd‐1* lines 8660‐6 and 8660‐12 along with a wild‐type control were transferred to MS media with or without 50 µm GA_3_, and plant height was recorded after 11 days. Data collected showed increases in stem elongation in these two lines over the untreated controls (Figure S3c,d). Both greenhouse and *in vitro* experiments clearly demonstrated that the dwarfing phenotype in *sd‐1* lines could be rescued by GA_3_ application.

### Estimation of lodging using image data

**Figure 7:**
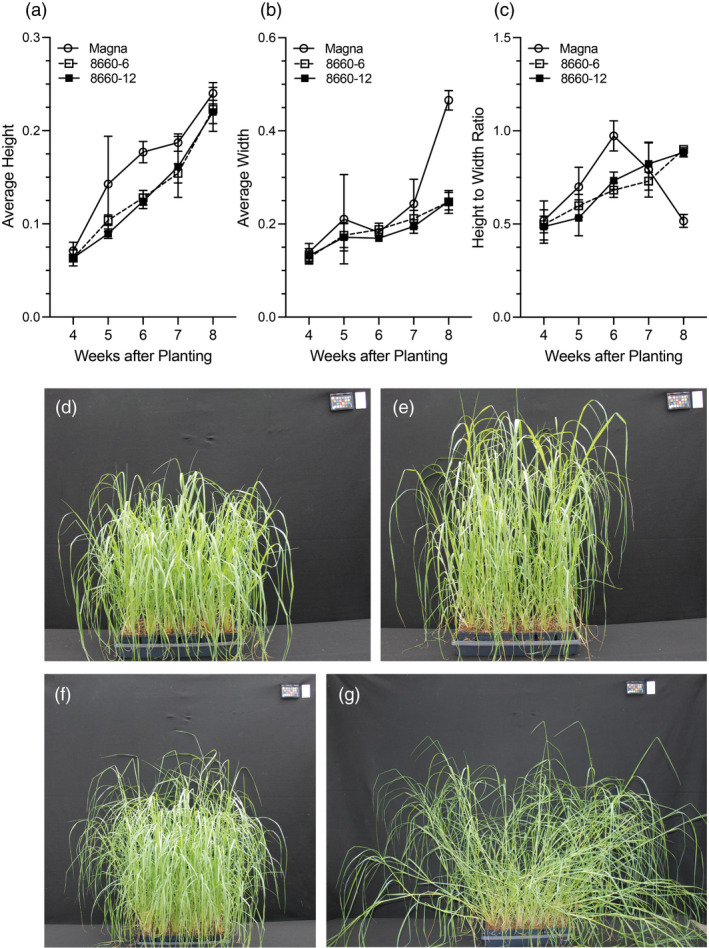
Tef lodging analyses using PlantCV. Tef plant pictures were collected on weekly basis from the 4th to 8th weeks after planting. Lodging was estimated by analyzing collected images using PlantCV from plant height, width and computing height to width ratio. Average normalized plant height (a); width (b) and plant height to width ratio (c) of two semidwarf *sd‐1* tef lines and the wild‐type Magna cultivar. Representative images of *sd‐1* tef line collected at 6 (d) and 8 (f), and wild‐type Magna at 6 (e) and 8 (g) weeks after planting. Note lodging in the wild‐type control.

Numerical estimation of lodging in tef was done by calculating the height : width ratios from tef images collected over time using PlantCV (Gehan *et al*., 2017). Using these images, the shape of the plant was identified, and a reference line was placed at the root/soil base for every image to generate a height above the reference line measurement. To compensate for the distance from the camera, the height data were normalized with the area of the size marker in the images. The height above the reference line for each image was divided by the area of the size marker, to obtain a normalized height. Across all tef entries, plant height increased over time (Figure 7a). The control Magna and edited *sd‐1* lines reached maximum height by 8 weeks after planting. Tef produces many stems (tillers). Due to differential curvature, they do not all lodge together. Height alone, therefore, cannot be used as a determinant of lodging, and hence, it must be combined with plant width. As for height measurements, the width measurements were normalized with the size marker area, and the averages were plotted (Figure 7b). Up to 6 weeks after planting, the Magna control and *sd‐1* edited lines exhibited similar increases in width, with the width of the Magna control significantly (*P* < 0.05) increasing after 7 weeks compared to that of the *sd‐1* lines (Figure 7b).

As indicated above, lodging includes changes in both the height and width of the plant. Therefore, the height : width ratio was calculated as an estimation of lodging (Figure 7c). In the *sd‐1* lines, the height : width ratio steadily increased over time. The control Magna had a greater increase in height : width ratio in comparison with *sd‐1* lines until 6 weeks after planting (Figure 7c) and thereafter decreased rapidly indicating a significant degree of lodging that was also confirmed by visually inspecting the image data (Figure 7e,g). In contrast, although the height : width ratio of the *SD‐1* edited lines increased over time (Figure 7c), there were no obvious visual indications of lodging (Figure 7d,f) during the study period.

### Measurement of lodging using USDA‐GRINS descriptor

A similar measurement of lodging at the heading stage, based on the USDA‐GRINS descriptor that measures lodging on the scale of 1 to 9 showed a significant (*P* < 0.01) difference between *sd‐1* lines, with an average score of 1.2–1.3, and the Magna control, with an average lodging score of 5 (Figure S4).

## Discussion

Successful production of gene‐edited plants requires a robust transformation and plant regeneration system that, to date, has been a limiting factor for tef. Recent advances in the use of the plant transcription factors *BBM* and *WUS2* (Gordon‐Kamm *et al*., 2019; Lowe *et al*., 2016, 2018) and the co‐expression of *GROWTH‐REGULATING FACTOR* 4 (*GRF4*), and its cofactor *GRF‐INTERACTING FACTOR 1* (*GIF1*; Debernardi *et al*., 2020), have shown significant improvements in genetic transformation and frequency of recovery of transgenic and gene‐edited events in otherwise recalcitrant monocot species.

Two prior reports showed successful transformation and regeneration of transgenic tef plants. A single transgenic tef plant expressing a reporter gene was first reported by Gugsa (2005) using an immature zygotic embryo as an explant. Gebre *et al*. (2013) also reported the generation of eight T_0_ transgenic tef lines expressing a GA 2‐oxidase, an enzyme that deactivates bioactive GAs or their precursors (Yamaguchi, 2008) using an immature zygotic embryo as an explant. The transformation system we developed for tef is advantageous over earlier methods due to (i) the use of readily available young leaf explants, which circumvents the need for extra space and time required to produce a continuous supply of flowering tef plants as a source of explants, (ii) avoiding the tedious dissection and collection of immature zygotic embryos from one of the smallest cereal seed, and (iii) the co‐expression of *BBM* and *WUS2* to induce *de novo* somatic embryogenesis, which was critical for the successful recovery of transformed and gene‐edited tef lines at a very high frequency. In our study, the transformation of tef using the young leaf as an explant was efficacious (Table S3) with constitutive expression of *BBM* and *WUS2*. These morphogenic genes were, however, known to have undesirable pleiotropic effects when expressed in regenerated plants, and finetuned expression or programmed excision of these genes have been reported to overcome such effects (Hoerster *et al*., 2020; Lowe *et al*., 2016, 2018; Wang *et al*., 2020). The construct p8702 had *Cre* expression driven by RAB17 promoter (Figure S2) for ABA or desiccation induced excision of these genes. Inclusion of ABA at 50 µm before regeneration was sufficient to induce the excision of these genes in our experiments.

Bioactive gibberellins (GA_1_ and GA_4_) are involved in diverse plant processes including seed germination, stem elongation, leaf expansion, floral transition and seed/fruit development (Achard and Genschik, 2009; Yamaguchi, 2008). Like rice and most other monocot species, tef has four GA 20‐oxidase genes (Figure 1). Though transgenic RNAi lines of rice *GA20ox1,* that predominantly expressed in reproductive organs, are known to cause semidwarfism (Oikawa *et al*., 2004) and RNAi lines of *GA20ox3* to cause semidwarf and disease resistance phenotype (Qin *et al*., 2013), only mutations in the *GA20ox2* gene (*sd‐1*) have been widely introgressed and utilized in rice production due to the beneficial effect on plant stature, lodging resistance, increased harvest index and responsiveness to nitrogen fertilizer application (Peng *et al*., 2021). Up to 10 alleles of *SD‐1*, obtained through spontaneous mutation or conventional mutagenesis, are known so far (Peng *et al*., 2021) and more alleles are being introduced through genome editing (Biswas *et al*., 2020; Hu *et al*., 2019).

Inspired by the wide application of the *SD‐1* mutation in rice to combat lodging through reduced height, we generated multiple tef lines with knockout mutations in tef *SD‐1* gene. Tef being a self‐pollinating allotetraploid (2*n* = 4× = 40; VanBuren *et al*., 2020), mutation of all four alleles of *SD‐1* gene was required to see the altered phenotype due to the recessive nature of *sd‐1* mutation (Hedden, 2003). Most of the tetra‐allelic mutations obtained in both tef cultivars in this study (Figure 3 and Table S4) were frameshift mutations resulting in the introduction of a premature termination codon.

Disruption of *SD‐1* function results in an increase in the level of GA_53_, (a GA intermediate and a substrate for GA 20‐oxidase) and reduced levels of bioactive GA_1_ precursor, GA_20_ and bioactive GA_1_ (Sasaki *et al*., 2002; Spielmeyer *et al*., 2002) leading to shorter internode and culm lengths as observed in *sd‐1* lines of indica (Chunhai and Zongtan, 1996) and japonica rice (Tomita and Ishii, 2018). Although we did not measure levels of bioactive GA in tef lines, the *sd‐1* tef lines have shown a reduction in culm length, due to the reduction in lengths of all internodes (internode‐2 to internode‐7; Figure 5e). This reduction in culm length (plant height) could be rescued by the application GA_3_ (Figure S3) indicating that the GA deficiency due to disruption of *SD‐1* gene was responsible for the observed semidwarfism in *sd‐1* tef lines. Like earlier findings in rice, we did not see significant differences in panicle lengths between *sd‐1* tef lines and the wild‐type control. The absence of negative impacts of the *sd‐1* mutation on panicle length could be due to redundancies of the GA 20‐oxidases (Figure 1), which were shown to have specificity in expression and function in different plant tissues and developmental stages (Sakamoto *et al*., 2004a, 2004b).

Three types of lodging are known in cereal crop plants: stem bending (leaning), stem buckling and root lodging (Hirano *et al*., 2017b). In tef, the predominant form of lodging is considered as stem bending (Bayable *et al*., 2020; Ketema, 1983), although Van Delden *et al*. (2010) reported that root lodging was the predominant type of lodging in combination with weaker stem strengths. Resistance to stem bending lodging is the type conferred by *SD‐1* mutation in rice (Hirano *et al*., 2017b). Under field conditions, lodging in tef is typically measured following the method of Caldicott and Nuttall (1979), but various methods have been described both for field and greenhouse‐grown plants (Bayable *et al*., 2020; Ben‐Zeev *et al*., 2020; Blösch *et al*., 2020; Van Delden *et al*., 2010). Since our study was based on potted plants in the greenhouse, we used lodging indices that measured the degree of leaning on a 1–9 rating based on USDA‐GRINS descriptors used for tef and also through time course image analysis using PlantCV functions (Gehan *et al*., 2017) that considered changes in plant shape based on height to width ratio over time. Based on observations from multiple greenhouse experiments lodging due to stem bending was the predominant type of lodging observed for the control Magna plants, which began during vegetative growth (5–6 weeks after planting) with most plants fully lodged by 8 weeks. Using both the USDA‐GRINS rating scale and image analysis, the *sd‐1* edited tef lines were found to be significantly more resistant to lodging than the unmodified control (Figures 7 and S4). These observations were made under greenhouse conditions with no wind and rain, and the outcome could be different under field conditions where the wetness of the soil and the plant either from rain or dew have been reported to affect lodging (Van Delden *et al*., 2010).

Tef’s morphological characteristics including plant height, lengths of the panicle, the peduncle, the internodes (culm length), thickness of the basal internodes, angle of the panicle, tiller number, root characteristics and the relationship of these plant parts with each other are known to influence the degree of lodging in tef (Bayable *et al*., 2020; Blösch *et al*., 2020; Girma, 2019; Ketema, 1983; Van Delden *et al*., 2010), besides environmental conditions and agronomic practices (Ben‐Zeev *et al*., 2020; Merchuk‐Ovnat *et al*., 2020). The reduction in culm length and increased thickness of the first and second basal internodes are likely factors for the increased lodging resistance observed for the *sd‐1* tef lines. Further studies under field conditions are necessary to confirm and validate the lodging resistance reported in this study.

## Conclusions

We developed a robust and reproducible tef transformation and CRISPR/Cas9 targeted gene editing system for this important food security crop and showed that an important trait such as lodging resistance can be achieved in a fraction of time and resources required by conventional plant breeding. We believe this will serve as an additional toolkit for breeders and product developers in their quest to improve tef to the benefit of Ethiopian farmers and tef producers elsewhere. We trust that improvement in lodging resistance in tef can facilitate a tremendous increase in tef productivity both directly through reducing losses due to lodging and indirectly through allowing a modernized tef production system including the use of yield‐enhancing inputs such as fertilizers and machine harvesting of tef.

## Materials and methods

### Tef GA 20‐oxidases and identification of Tef SD‐1

Sequences of rice (*Oryza sativa* L.) GA 20‐oxidases (*OsGA20ox1‐OsGA20ox4*; Sakamoto *et al*., 2004a, 2004b) were used as baits to retrieve orthologous GA 20‐oxidases from the published tef genome (VanBuren *et al*., 2020) using CoGeBlast (Lyons *et al*., 2008). To construct a phylogenetic tree, the coding sequences (CDS) of GA 20‐oxidases of both tef and rice were analyzed with the phylogeny.fr (http://www.phylogeny.fr/, accessed June 15, 2021) pipeline using the default ‘One Click mode’ (Dereeper *et al*., 2008). The One Click mode uses ‘optimized programs including MUSCLE (Edgar, 2004) for multiple alignments, Gblocks (Castresana, 2000) for alignment curation, PhyML (Guindon and Gascuel, 2003) for phylogeny and finally TreeDyn (Chevenet *et al*., 2006) for tree drawing to reconstruct a phylogenetic tree from tef and rice GA 20‐oxidases’. Detailed descriptions of the programs are found at http://www.phylogeny.fr/.

### Guide RNA (gRNA) design and constructs

Two guide RNAs (gRNA1 and gRNA2) each targeting both homoeoalleles of the tef *SD‐1* gene were designed using the CRISPOR program (Haeussler *et al*., 2016). The designed gRNA oligonucleotides were synthesized at Integrated DNA Technologies (Coralville, IA) after introducing 5'‐compatible overhangs on forward and reverse gRNA oligos (Table S5). Assembly of the gRNA oligos into an expression system and subsequently into binary vectors followed the method described by Čermák *et al*. (2017). Briefly, the gRNA oligonucleotides were cloned into modular vectors pMOD_B2518 for gRNA1 and pMOD_C2517 for gRNA2. The two gRNA expression cassettes plus monocot codon‐optimized Cas9 (pMOD_A1110) and a binary vector harbouring hygromycin phosphotransferase II (pTRANS_250d) were assembled using Golden Gate (Engler *et al*., 2008) and cloned in MAX Efficiency™ DH5α competent cells (Thermo Fisher Scientific, Waltham, MA). The cloned gRNA oligonucleotides and Cas9 were confirmed through sequencing using primers listed in Table S5. The resulting binary vector was named p8603. From this binary vector, the *Zm‐Ubi_pro_*::*Cas9*// *Ta‐U6_pro_*::*EtSD1sgRNA1*// *Ta‐U3_pro_*::*EtSD1sgRNA2* cassette was excised as an *Asc*I‐*Pme*I fragment (*Asc*I site blunted) that was cloned into the *Swa*I site of PHP81814 to generate p8660, and into the *Fse*I site of the binary vector PHP78891 (after blunting) to generate p8702. The two recipient binary vectors, PHP81814 and PHP78891 harbour the morphogenic regulator genes from maize, *BBM* and *WUS2,* and a green fluorescent protein (GFP) marker gene ZsGreen (Lowe *et al*., 2016). The binary vectors p8660 and p8702 (Figures S1 and S2) were electroporated into thymidine auxotrophic (THY‐) LBA4404 *Agrobacterium tumefaciens* (Lowe *et al*., 2016) and used for tef transformation. Binary vectors PHP81814 and PHP78891 and *A*. *tumefaciens* strain LBA4404 THY‐ harbouring the helper plasmid PHP71539 were kindly provided by Corteva Agriscience, Johnston, IA.

### Transformation and regeneration of tef lines

#### Seedling and leaf explant preparation

Seeds of tef cultivars Magna, and Ada, accession numbers, PI 243908 and PI 524433, respectively, were obtained from the National Genetic Resources Program of the United State Department of Agriculture (USDA). Seeds were rinsed with distilled water, sterilized by agitating in 50% (v/v) commercial bleach containing 1–2 drops of Tween‐20 for 10 min and rinsed five times with sterile distilled water. Between 12 and 15 seeds were germinated per petri dish, containing Murashige and Skoog (MS) basal medium (Murashige and Skoog, 1962) supplemented with 20 g/L sucrose (MS2) and 2.2 g/L gelzan. A sterile inverted sundae cup (https://www.webstaurantstore.com, item # 760SD12) was placed over the petri dish bottom, sealed and cultured at 28 °C for 10–14 days in a walk‐in growth room maintained at 16 h day and 8 h dark with light intensity of 90 µmol/m^2^/s. After 10–14 days shoots were cut approximately 0.25 cm above the base using a sterile scalpel blade and the upper portion of leaves (above the topmost collar) was removed. The stem base consisting of folded young leaves was sectioned transversely to generate shoot material approximately 0.4–0.8 cm in length. The stem material was sliced longitudinally to generate two similar‐sized explants per section and used for *Agrobacterium* inoculation.

#### *Agrobacterium* preparation and explant inoculation

The *Agrobacterium* LBA4404 THY‐ harbouring the binary vectors p8660 and p8702 was streaked on Luria broth (LB) containing 50 mg/L thymidine, 50 mg/L gentamicin and 100 mg/L spectinomycin to produce a master plate and cultured for 3–4 days at 28 °C and used as a master plate for up to 3 weeks by storing at 4 °C. One day prior to transformation, a fresh plate was re‐streaked from the master plate and cultured at 28 °C for 24 h. On the day of transformation, the *Agrobacterium* culture was scraped from the medium and transferred to a liquid inoculation medium consisting of MS basal medium supplemented with 68.5 g/L sucrose, 36 g/L glucose, 6.79 µm 2,4‐D, 100 µm acetosyringone and 0.02% (v/v) Alligare Surface nonionic surfactant (https://alligare.com). The *Agrobacterium* suspension was vortexed and adjusted to an OD_600_ = 0.5. Three millilitres of inoculation medium were pipetted into each well of a 6‐well plate, and 25–30 explants were added to each well and vacuum infiltrated for 30 min. Explants were then removed using fine forceps, briefly touched onto a sterile filter to draw off excess inoculation medium and placed into Petri dishes containing MS2 medium supplemented with 10 g/L glucose, 10 µm 2,4‐D, 100 µm acetosyringone and 50 mg/L thymidine and solidified with 4 g/L gelzan. Co‐culture was performed for 3 days at 22 °C in the dark, after which explants were transferred to a callus induction medium consisting of MS basal medium supplemented with 30 g/L sucrose (MS3), 10 µm 2,4‐D, 100 mg/L cefotaxime and 4 g/L gelzan and cultured for 4 weeks in the dark at 28 °C.

#### Selection of transgenic tissues and plant regeneration

Green fluorescent protein fluorescing callus units were observed 3 days post‐inoculation and monitored on a weekly basis. Starting after 4 weeks, GFP fluorescing callus units were transferred onto a excision medium consisting of either MS3 supplemented with 10 µm 2,4‐D and 50 µm abscisic acid (ABA), for tissues transformed with p8702 and cultured for 48 h in the dark at 28 °C followed by a transfer onto the regeneration medium. Tissues transformed with p8660 were subcultured directly to regeneration medium, consisting of MS3 supplemented with 6 µm 6‐benzylaminopurine (BAP). All tissues were cultured on a regeneration medium for 3–4 weeks in a 16 h light 8 h dark cycle. Greening shootlets developing from the callus were transferred onto MS3 supplemented with 2 µm BAP and cultured for 3–4 weeks in 16 h light until individual shoots were regenerated. These were separated and transferred onto MS2 gelzan for root initiation and growth with an inverted sundae cup dome placed over the petri dish bottom.

### Plant growth conditions

Rooted *in vitro* plantlets or tef seeds were planted in 3.5‐inch pots containing Jolly Gardener Pro‐Line C/V potting mix and grown in a walk‐in growth chamber maintained at 29/22 °C day/night temperature and relative humidity (RH) of 50% with 12 h light at 500 µmol/m^2^/s. Greenhouse conditions were 29/22 °C day/night temperature and RH of about 50%. Day length was not controlled in the greenhouse. Tef seeds were planted in potting media by distributing (about 10–20 seeds per pot) on top of potting mix and then lightly covering with a 0.1–0.2 cm layer of potting mix. The potting mix was wetted with water before and after planting and placed in 18 pot‐holding trays covered with transparent plastic domes. The domes were removed 5 days later, and the seedlings were thinned/transplanted to one plant per pot 8–10 days after sowing (three‐leaf stage) and the plants were grown to maturity in a growth chamber or greenhouse. The plants were staked as necessary. Growth chamber and greenhouse‐grown plants were fertilized three times a week with water‐soluble fertilizers at a rate of 175–200 ppm using either Jack’s 15‐5‐15 Ca‐Mg LX or 15‐16‐17 Peat‐Lite, respectively.

### Molecular characterization

To identify *SD‐1* gene mutations, genomic DNA was extracted from leaf tissues of T_0,_ T_1_ and T_2_ plants as described previously (Gao *et al*., 2010) or using Qiagen DNeasy plant mini kit (Qiagen, Germantown, MD). The tef genome is an allotetraploid (2*C* = 2*n* = 4× = 40) consisting of two diploid subgenomes, designated as the A and B genomes. Amplicons of 117 and 97 bp, and 140 and 107 bp, are expected for the *SD‐1* gRNA1 target site (TS1) and *SD‐1* gRNA2 TS2 in the A and B genomes, respectively. Next‐generation sequencing was used to evaluate the target site sequence changes. Target sequences were amplified first with Phusion Flash High Fidelity PCR Master Mix (F‐548; Thermo Fisher Scientific), then secondary amplification used Phusion Master Mix F‐531 (Thermo Fisher Scientific) following a standard PCR protocol. The Molecular Inversion Probe (MIP) primers used are listed in Table S6. The resulting PCR products were purified with a Qiagen PCR purification spin column, DNA concentrations measured with a Hoechst dye‐based fluorometric assay and then sequenced directly with next‐generation sequencing. To detect genome editing reagents and helper genes in the T_1_ and T_2_ generations, qPCR was performed using Qiagen QuantiTect Multiplex PCR Master Mix. The primers and probes used are listed in Table S7. The tef phytoene desaturase (PDS) gene was used as an internal control for PCR.

### Measurements of morphological traits on T_1_ and T_2_ sd‐1 plants

Morphological traits, including plant height, panicle, peduncle, internode lengths and tiller number were recorded at harvest as described earlier (Ketema, 1983). Briefly, panicle length was measured from the base of the inflorescence (beginning from the base of the lowest panicle branch to the tip of the panicle), and peduncle length (also called internode 1 in this study) was measured from the topmost node on the culm to the base of the inflorescence. Internode length was measured as the length between two consecutive nodes, with internode number 2 being the topmost after the peduncle, with increasing internode number towards the base of the plant. Culm length was determined by adding the lengths of all internodes other than peduncle and panicle. Plant height data were obtained by adding culm, peduncle and panicle lengths. For data collected at harvest, measurements were collected from ten plants per line and three stems per plant (pot). Plant height data were also collected 5 weeks after seed planting from 18–36 main stems by measuring stem height from the base at the soil surface to the topmost collar.

### Measurements of lodging resistance

A total of three entries consisting of, two T_2_ generation of *SD‐1* knockout lines in Magna cultivar background and a wild‐type control, were used for assessing lodging resistance. The *SD‐1* edited lines and the control plants were planted in three trays each with 18 plants per tray and grown in the greenhouse. Assessments of lodging were made at the heading stage (8 weeks after planting) based on individual pots from three replicated trays (total of 54 potted plants per line). Two methods were used for measuring resistance to lodging. The first used the USDA‐GRINS morphological descriptors for cool‐season grasses, including tef, where lodging was scored on a 1–9 scale, with 1 being no lodging and 9 being extreme lodging (https://npgsweb.ars‐grin.gov/gringlobal/descriptordetail?id=110126). The second method employed image analysis technology. Images of tef plants in trays were collected at weekly intervals during the vegetative growth phase (weeks 4–8 after planting) using a Nikon D80 camera. Collected images were then analyzed using PlantCV (Gehan *et al*., 2017). Using PlantCV, tef plants were segmented from background objects and the ‘analyze_shape’ function was used to measure the width of the isolated tef plant images, while the ‘analyze_bound_horizontal’ function was used to set a reference line at the top of the pot‐holding tray in order to estimate plant height. A size marker within the image was measured to normalize values since there could be a slight variation in camera position. The height, width and height_above_horizontal_bound/width ratio were used to estimate lodging.

### GA_3_ treatment

GA_3_ (Catalog No. 7645, Sigma Aldrich, St. Louis, MO, USA) was used at final concertation of 50 µm prepared in Milli‐Q water containing 0.1% (v/v) Surface (https://alligare.com) for greenhouse spray treatment or included in MS2 media containing 2.2 g/L gelzan. The untreated controls were Milli‐Q water containing 0.1% (v/v) Surface without GA_3_ for greenhouse spray and MS2 media with 2.2 g/L gelzan without GA_3_. For greenhouse GA spray treatment, 6‐week‐old plants were used while for *in vitro* GA treatment, *in vitro* germinated 6‐day‐old seedlings were used. GA treatment lasted for 11 days after which plants were scored for stem elongation (plant height).

### Data analysis

Plant morphological data including plant height, lengths of culm, internodes, panicle and peduncle were analyzed by one‐way analysis of variance and Dunnett’s multiple comparisons test (comparison of means of edited events with wild‐type control) using GraphPad Prism version 9.1.2 for macOS, GraphPad Software, San Diego, CA. Lodging data (height/width ratio) were analyzed using R (R Core Team, 2013).

## Conflict of interest

The authors declare no conflicts of interest.

## Authors contributions

GB, DJM, NJT, EG, DG, SC, KA and GT conceived the experiment. GB, DJM, NJT, RDC, JV, NH, NW, MG, MM, MY, BL, CS, HG and WG‐K designed the experiments and generated the data. GB, DJM, HG and MG analyzed the data. GB and DJM wrote the manuscript. All authors reviewed and commented on the manuscript.

## Supporting information


**Figure S1** Plasmid maps of p8660 used to generate transgenic and gene‐edited tef lines.
**Figure S2** Plasmid map of p8702 used to generate transgenic and gene‐edited tef lines.
**Figure S3** GA_3_ treatment effect on stem elongation in the greenhouse‐grown (a, b) and in vitro grown (c, d) plants of *SD‐1* knockout lines (8660‐6, 8660‐12) and wild‐type control.
**Figure S4** Lodging in *sd‐1* tef line and wild‐type Magna control.

**Table S1** Nucleotide sequence identity among rice and tef GA 20‐oxidases
**Table S2** Amino acid sequence identity among rice and tef GA 20‐oxidases
**Table S3** Transformation efficiency in two tef cultivars using young leaf explants
**Table S4** *SD‐1* mutation detected at target site 1 and target site 2 in both subgenome‐A and subgenome‐B
**Table S5** Sequences of guide RNAs (gRNAs) oligos targeting tef *SD‐1* and sequencing primers for confirmation of cloned gRNAs and Cas9
**Table S6** Primers used to generate target site amplicons for Next‐Generation Sequencing (NGS) of *SD‐1* gene mutation
**Table S7** Quantitative PCR (qPCR) primers used to detect T‐DNA in T1/T2 plants

## Data Availability

Novel biological materials described in this publication may be available to academic requesters and other not‐for‐profit institutions solely for noncommercial research purposes upon acceptance and signing of a material transfer agreement between the requester institution and the applicable author institution for the particular materials. In some cases, such materials may originally contain genetic elements described in the manuscript that was obtained from a third party(s), and the authors may not be able to provide materials including third party genetic elements to the requestor because of certain third party contractual restrictions placed on the author's institution. In such cases, the requester will be required to obtain such materials directly from the third party. The authors and authors' institutions do not make any express or implied permission(s) to the requester to make, use, sell, offer for sale, or import third party proprietary materials. Obtaining any such permission(s) will be the sole responsibility of the requestor. Corteva Agriscience *Agrobacterium* strain, genetic elements and plasmids for making transgenic or edited plants would be available to academic requesters and other not‐for‐profit institutions solely for noncommercial research purposes upon acceptance and signing of a material transfer agreement with stewardship requirements.

## References

[pbi13842-bib-0001] Achard, P. and Genschik, P. (2009) Releasing the brakes of plant growth: how GAs shutdown DELLA proteins. J. Exp. Bot. 60, 1085–1092. 19043067 10.1093/jxb/ern301

[pbi13842-bib-0002] Asano, K. , Yamasaki, M. , Takuno, S. , Miura, K. , Katagiri, S. , Ito, T. , Doi, K. *et al*. (2011) Artificial selection for a green revolution gene during japonica rice domestication. Proc. Natl. Acad. Sci. USA, 108, 11034–11039. 21646530 10.1073/pnas.1019490108 PMC3131315

[pbi13842-bib-0003] Ashikari, M. , Sasaki, A. , Ueguchi‐Tanaka, M. , Itoh, H. , Nishimura, A. , Datta, S. , Ishiyama, K. *et al*. (2002) Loss‐of‐function of a rice gibberellin biosynthetic gene, GA20 oxidase (GA20ox‐2), led to the rice ‘green revolution’. Breed. Sci. 52, 143–150.

[pbi13842-bib-0004] Assefa, K. , Cannarozzi, G. , Girma, D. , Kamies, R. , Chanyalew, S. , Plaza‐Wuthrich, S. , Blosch, R. *et al*. (2015) Genetic diversity in tef [*Eragrostis tef* (Zucc.) Trotter]. Front. Plant Sci. 6, 1–13. 25859251 10.3389/fpls.2015.00177 PMC4374454

[pbi13842-bib-0005] Assefa, K. and Chanyalew, S. (2018) Agronomics of tef. In Minten, B., Taffesse, A.S. and Brown, P. eds., The Economics of Tef, Exploring Ethiopia’s Biggest Cash Crop, pp. 39–70. Wasington, DC: International Food Policy Research Institute (IFPRI).

[pbi13842-bib-0006] Assefa, K. , Ketema, S. , Tefera, H. , Nguyen, H.T. , Blum, A. , Ayele, M. , Bai, G. *et al*. (1999) Diversity among germplasm lines of the Ethiopian cereal tef [*Eragrostis tef* (Zucc.) Trotter]. Euphytica, 106, 87–97.

[pbi13842-bib-0007] Assefa, K. , Yu, J. , Zeid, M. , Belay, G. , Tefera, H. and Sorrells, M.E. (2011) Breeding tef [*Eragrostis tef* (Zucc.) trotter]: conventional and molecular approaches. Plant Breed. 130, 1–9.

[pbi13842-bib-0008] Bayable, M. , Tsunekawa, A. , Haregeweyn, N. , Ishii, T. , Alemayehu, G. , Tsubo, M. , Adgo, E. *et al*. (2020) Biomechanical properties and agro‐morphological traits for improved lodging resistance in ethiopian teff (*Eragrostis tef* (Zucc.) Trottor) accessions. Agronomy, 10, 1012.

[pbi13842-bib-0009] Baye, K. (2014) Teff: Nutrient Composition and Health Benefits. ESSP Working Paper 67. Washington, D.C. and Addis Ababa, Ethiopia: International Food Policy Research Institute (IFPRI) and Ethiopian Development Research Institute (EDRI).

[pbi13842-bib-0010] Bekana, G. and Assefa, K. (2021) Genetic variation, heritability and genetic advance among semi‐dwarf tef [*Eragrostis tef* (Zucc.) Trotter] recombinant inbred lines with emphasis to lodging. Am. J. Life Sci. 9, 92–104.

[pbi13842-bib-0011] Ben‐Zeev, S. , Rabinovitz, O. , Orlov‐Levin, V. , Chen, A. , Graff, N. , Goldwasser, Y. and Saranga, Y. (2020) Less is more: lower sowing rate of irrigated tef (*Eragrostis tef*) alters plant morphology and reduces lodging. Agronomy, 10, 570.

[pbi13842-bib-0012] Biswas, S. , Tian, J. , Li, R. , Chen, X. , Luo, Z. , Chen, M. , Zhao, X. *et al*. (2020) Investigation of CRISPR/Cas9‐induced SD1 rice mutants highlights the importance of molecular characterization in plant molecular breeding. J. Genet. Genomics, 47, 273–280. 32684419 10.1016/j.jgg.2020.04.004

[pbi13842-bib-0013] Blösch, R. , Plaza‐Wüthrich, S. , Barbier de Reuille, P. , Weichert, A. , Routier‐Kierzkowska, A.‐L. , Cannarozzi, G. , Robinson, S. *et al*. (2020) Panicle angle is an important factor in tef lodging tolerance. Front. Plant Sci. 11, 61. 32117397 10.3389/fpls.2020.00061 PMC7031273

[pbi13842-bib-0014] Caldicott, J.J.B. and Nuttall, A. (1979) A method for the assessment of lodging in cereal crops. J. Natl. Inst. Agric. Bot. 15, 88–91.

[pbi13842-bib-0015] Castresana, J. (2000) Selection of conserved blocks from multiple alignments for their use in phylogenetic analysis. Mol. Biol. Evol. 17, 540–552. 10742046 10.1093/oxfordjournals.molbev.a026334

[pbi13842-bib-0016] Čermák, T. , Curtin, S.J. , Gil‐Humanes, J. , Čegan, R. , Kono, T.J.Y. , Konečná, E. , Belanto, J.J. *et al*. (2017) A multipurpose toolkit to enable advanced genome engineering in plants. Plant Cell, 29, 1196–1217. 28522548 10.1105/tpc.16.00922 PMC5502448

[pbi13842-bib-0017] Chevenet, F. , Brun, C. , Bañuls, A.‐L. , Jacq, B. and Christen, R. (2006) TreeDyn: towards dynamic graphics and annotations for analyses of trees. BMC Bioinformatics, 7, 1–9. 17032440 10.1186/1471-2105-7-439 PMC1615880

[pbi13842-bib-0018] Chono, M. , Honda, I. , Zeniya, H. , Yoneyama, K. , Saisho, D. , Takeda, K. , Takatsuto, S. *et al*. (2003) A semidwarf phenotype of barley uzu results from a nucleotide substitution in the gene encoding a putative brassinosteroid receptor. Plant Physiol. 133, 1209–1219. 14551335 10.1104/pp.103.026195 PMC281616

[pbi13842-bib-0019] Chunhai, S. and Zongtan, S. (1996) Effects of semidwarf gene sd1 on agronomic traits in rice (*Oryza sativa* subsp. indica). Zhongguo Shuidao Kexue, 10, 13–18.

[pbi13842-bib-0020] Clasen, B.M. , Stoddard, T.J. , Luo, S. , Demorest, Z.L. , Li, J. , Cedrone, F. , Tibebu, R. *et al*. (2016) Improving cold storage and processing traits in potato through targeted gene knockout. Plant Biotechnol. J. 14, 169–176. 25846201 10.1111/pbi.12370 PMC11389148

[pbi13842-bib-0021] Cochrane, L. and Bekele, Y.W. (2018) Average crop yield (2001–2017) in Ethiopia: trends at national, regional and zonal levels. Data Br. 16, 1025. 10.1016/j.dib.2017.12.039 PMC5758922 29326965

[pbi13842-bib-0022] Debernardi, J.M. , Tricoli, D.M. , Ercoli, M.F. , Hayta, S. , Ronald, P. , Palatnik, J.F. and Dubcovsky, J. (2020) A GRF–GIF chimeric protein improves the regeneration efficiency of transgenic plants. Nat. Biotechnol. 38, 1274–1279. 33046875 10.1038/s41587-020-0703-0 PMC7642171

[pbi13842-bib-0023] Demeke, M. and Di Marcantonio, F. (2019) Analysis of incentives and disincentives for teff in Ethiopia. Gates Open Res. 3, 212.

[pbi13842-bib-0024] Dereeper, A. , Guignon, V. , Blanc, G. , Audic, S. , Buffet, S. , Chevenet, F. , Dufayard, J.F. *et al*. (2008) Phylogeny.fr: robust phylogenetic analysis for the non‐specialist. Nucleic Acids Res. 36, 465–469. 10.1093/nar/gkn180 PMC2447785 18424797

[pbi13842-bib-0025] Edgar, R.C. (2004) MUSCLE: multiple sequence alignment with high accuracy and high throughput. Nucleic Acids Res. 32, 1792–1797. 15034147 10.1093/nar/gkh340 PMC390337

[pbi13842-bib-0026] Engler, C. , Kandzia, R. and Marillonnet, S. (2008) A one pot, one step, precision cloning method with high throughput capability. PLoS ONE, 3, e3647. 18985154 10.1371/journal.pone.0003647 PMC2574415

[pbi13842-bib-0027] Farrow, S.C. and Facchini, P.J. (2014) Functional diversity of 2‐oxoglutarate/Fe (II)‐dependent dioxygenases in plant metabolism. Front. Plant Sci. 5, 524. 25346740 10.3389/fpls.2014.00524 PMC4191161

[pbi13842-bib-0028] Gao, H. , Gadlage, M.J. , Lafitte, H.R. , Lenderts, B. , Yang, M. , Schroder, M. , Farrell, J. *et al*. (2020) Superior field performance of waxy corn engineered using CRISPR–Cas9. Nat. Biotechnol. 38, 579–581. 32152597 10.1038/s41587-020-0444-0

[pbi13842-bib-0029] Gao, H. , Smith, J. , Yang, M. , Jones, S. , Djukanovic, V. , Nicholson, M.G. , West, A. *et al*. (2010) Heritable targeted mutagenesis in maize using a designed endonuclease. Plant J. 61, 176–187. 19811621 10.1111/j.1365-313X.2009.04041.x

[pbi13842-bib-0030] Gebre, E. , Gugsa, L. , Schlüter, U. and Kunert, K. (2013) Transformation of tef (*Eragrostis tef*) by *Agrobacterium* through immature embryo regeneration system for inducing semi‐dwarfism. S. Afr. J. Bot. 87, 9–17.

[pbi13842-bib-0031] Gebre, E. , Schlüter, U. , Hedden, P. and Kunert, K. (2012) Gibberellin biosynthesis inhibitors help control plant height for improving lodging resistance in *E. tef* (*Eragrostis tef*). J. Crop Improv. 26, 375–388.

[pbi13842-bib-0032] Gehan, M.A. , Fahlgren, N. , Abbasi, A. , Berry, J.C. , Callen, S.T. , Chavez, L. , Doust, A.N. *et al*. (2017) PlantCV v2: image analysis software for high‐throughput plant phenotyping. PeerJ, 5, e4088. 29209576 10.7717/peerj.4088 PMC5713628

[pbi13842-bib-0089] Girma, D. (2019) The Relationships between Stem Characters and Lodging Tolerance in Tef (Eragrostis tef) Genotypes. Ethiop. J., Agric. Sci. 29, 59–70.

[pbi13842-bib-0033] Gomez, M.A. , Lin, Z.D. , Moll, T. , Chauhan, R.D. , Hayden, L. , Renninger, K. , Beyene, G. *et al*. (2019) Simultaneous CRISPR/Cas9‐mediated editing of cassava eIF 4E isoforms nCBP‐1 and nCBP‐2 reduces cassava brown streak disease symptom severity and incidence. Plant Biotechnol. J. 17, 421–434. 30019807 10.1111/pbi.12987 PMC6335076

[pbi13842-bib-0034] Gordon‐Kamm, B. , Sardesai, N. , Arling, M. , Lowe, K. , Hoerster, G. , Betts, S. and Jones, T. (2019) Using morphogenic genes to improve recovery and regeneration of transgenic plants. Plants, 8, 38. 30754699 10.3390/plants8020038 PMC6409764

[pbi13842-bib-0035] Gugsa, L.T. (2005) Biotechnological studies in tef [*Eragrostis tef* (Zucc.) Trotter] with reference to embryo rescue, plant regeneration, haplodization and genetic transformation. PhD Thesis. Addis Ababa University and University of Humburg.

[pbi13842-bib-0036] Guindon, S. and Gascuel, O. (2003) A simple, fast, and accurate algorithm to estimate large phylogenies by maximum likelihood. Syst. Biol. 52, 696–704. 14530136 10.1080/10635150390235520

[pbi13842-bib-0037] Haeussler, M. , Schönig, K. , Eckert, H. , Eschstruth, A. , Mianné, J. , Renaud, J.‐B. , Schneider‐Maunoury, S. *et al*. (2016) Evaluation of off‐target and on‐target scoring algorithms and integration into the guide RNA selection tool CRISPOR. Genome Biol. 17, 148. 27380939 10.1186/s13059-016-1012-2 PMC4934014

[pbi13842-bib-0038] Haun, W. , Coffman, A. , Clasen, B.M. , Demorest, Z.L. , Lowy, A. , Ray, E. , Retterath, A. *et al*. (2014) Improved soybean oil quality by targeted mutagenesis of the fatty acid desaturase 2 gene family. Plant Biotechnol. J. 12, 934–940. 24851712 10.1111/pbi.12201

[pbi13842-bib-0039] Hedden, P. (2003) The genes of the Green Revolution. Trends Genet. 19, 5–9. 12493241 10.1016/s0168-9525(02)00009-4

[pbi13842-bib-0040] Hilley, J.L. , Weers, B.D. , Truong, S.K. , McCormick, R.F. , Mattison, A.J. , McKinley, B.A. , Morishige, D.T. *et al*. (2017) Sorghum Dw2 encodes a protein kinase regulator of stem internode length. Sci. Rep. 7, 1–14. 28676627 10.1038/s41598-017-04609-5 PMC5496852

[pbi13842-bib-0041] Hirano, K. , Kawamura, M. , Araki‐Nakamura, S. , Fujimoto, H. , Ohmae‐Shinohara, K. , Yamaguchi, M. , Fujii, A. *et al*. (2017a) Sorghum DW1 positively regulates brassinosteroid signaling by inhibiting the nuclear localization of BRASSINOSTEROID INSENSITIVE 2. Sci. Rep. 7, 1–10. 28273925 10.1038/s41598-017-00096-w PMC5428356

[pbi13842-bib-0042] Hirano, K. , Ordonio, R.L. and Matsuoka, M. (2017b) Engineering the lodging resistance mechanism of post‐Green Revolution rice to meet future demands. Proc. Jpn. Acad. Ser. B, 93, 220–233. 28413198 10.2183/pjab.93.014 PMC5489430

[pbi13842-bib-0043] Hoerster, G. , Wang, N. , Ryan, L. , Wu, E. , Anand, A. , McBride, K. , Lowe, K. *et al*. (2020) Use of non‐integrating Zm‐Wus2 vectors to enhance maize transformation. In Vitro Cell. Dev. Biol. Plant, 56, 265–279.

[pbi13842-bib-0044] Hu, X. , Cui, Y. , Dong, G. , Feng, A. , Wang, D. , Zhao, C. , Zhang, Y.U. *et al*. (2019) Using CRISPR‐Cas9 to generate semi‐dwarf rice lines in elite landraces. Sci. Rep. 9, 1–7. 31836812 10.1038/s41598-019-55757-9 PMC6910903

[pbi13842-bib-0045] Jia, Q. , Li, C. , Shang, Y. , Zhu, J. , Hua, W. , Wang, J. , Yang, J. and *et al*. (2015) Molecular characterization and functional analysis of barley semi‐dwarf mutant Riso no. 9265. BMC Genom. 16, 1–11. 10.1186/s12864-015-2116-x PMC4647591 26573602

[pbi13842-bib-0046] Jöst, M. , Esfeld, K. , Burian, A. , Cannarozzi, G. , Chanyalew, S. , Kuhlemeier, C. , Assefa, K. *et al*. (2015) Semi‐dwarfism and lodging tolerance in tef (*Eragrostis tef*) is linked to a mutation in the α‐Tubulin 1 gene. J. Exp. Bot. 66, 933–944. 25399019 10.1093/jxb/eru452 PMC4321551

[pbi13842-bib-0047] Ketema, S. (1983) Studies of lodging, floral biology and breeding techniques in tef (*Eragrostis tef* (Zucc.) Trotter). PhD Thesis. University of London, UK.

[pbi13842-bib-0048] Ketema, S. (1991) Germplasm evaluation and breeding work on tef (*Eragrostis tef*) in Ethiopia. In Engels, J.M.M., Hawkes, J.G. and Worede, M. eds., *Plant Gentic Resources of Ethiopia*, pp. 323‐328. Cambridge University Press, Cambridge, New York, Port Chester, USA.

[pbi13842-bib-0049] Ketema, S. (1993) Tef (*Eragrostis tef*). breeding, agronomy, genetic resources, utilization and role in Ethiopian agriculture. Institute of Agricultural Research, Addis Ababa, Ethiopia.

[pbi13842-bib-0050] Liu, F. , Wang, P. , Zhang, X. , Li, X. , Yan, X. , Fu, D. and Wu, G. (2018) The genetic and molecular basis of crop height based on a rice model. Planta, 247, 1–26. 29110072 10.1007/s00425-017-2798-1

[pbi13842-bib-0051] Liu, L. , Gallagher, J. , Arevalo, E.D. , Chen, R. , Skopelitis, T. , Wu, Q. , Bartlett, M. *et al*. (2021) Enhancing grain‐yield‐related traits by CRISPR–Cas9 promoter editing of maize CLE genes. Nat. Plants, 7, 287–294. 33619356 10.1038/s41477-021-00858-5

[pbi13842-bib-0052] Lowe, K. , La Rota, M. , Hoerster, G. , Hastings, C. , Wang, N. , Chamberlin, M. , Wu, E. *et al*. (2018) Rapid genotype “independent” *Zea mays* L. (maize) transformation via direct somatic embryogenesis. In Vitro Cell. Dev. Biol. 54, 240–252. 10.1007/s11627-018-9905-2 PMC5954046 29780216

[pbi13842-bib-0053] Lowe, K. , Wu, E. , Wang, N. , Hoerster, G. , Hastings, C. , Cho, M.‐J. , Scelonge, C. *et al*. (2016) Morphogenic regulators Baby boom and Wuschel improve monocot transformation. Plant Cell, 28, 1998–2015. 27600536 10.1105/tpc.16.00124 PMC5059793

[pbi13842-bib-0054] Lyons, E. , Pedersen, B. , Kane, J. , Alam, M. , Ming, R. , Tang, H. , Wang, X. *et al*. (2008) Finding and comparing syntenic regions among Arabidopsis and the outgroups papaya, poplar, and grape: CoGe with rosids. Plant Physiol. 148, 1772–1781. 18952863 10.1104/pp.108.124867 PMC2593677

[pbi13842-bib-0055] Madeira, F. , Park, Y.M. , Lee, J. , Buso, N. , Gur, T. , Madhusoodanan, N. , Basutkar, P. *et al*. (2019) The EMBL‐EBI search and sequence analysis tools APIs in 2019. Nucleic Acids Res. 47, W636–W641. 30976793 10.1093/nar/gkz268 PMC6602479

[pbi13842-bib-0056] Merchuk‐Ovnat, L. , Bimro, J. , Yaakov, N. , Kutsher, Y. , Amir‐Segev, O. and Reuveni, M. (2020) In‐depth field characterization of teff [*Eragrostis tef* (Zucc.) Trotter] variation: from agronomic to sensory traits. Agronomy, 10, 1107.

[pbi13842-bib-0057] Minten, B. , Taffesse, A.S. and Brown, P. (2018) The economics of teff: exploring Ethiopia’s biggest cash crop. Washington, DC: International Food Policy Research Institute (IFPRI).

[pbi13842-bib-0058] Multani, D.S. , Briggs, S.P. , Chamberlin, M.A. , Blakeslee, J.J. , Murphy, A.S. and Johal, G.S. (2003) Loss of an MDR transporter in compact stalks of maize br2 and sorghum dw3 mutants. Science, 302, 81–84. 14526073 10.1126/science.1086072

[pbi13842-bib-0059] Murashige, T. and Skoog, F. (1962) A revised medium for rapid growth and bio assays with tobacco tissue cultures. Physiol. Plant, 15, 473–497.

[pbi13842-bib-0060] Oikawa, T. , Koshioka, M. , Kojima, K. , Yoshida, H. and Kawata, M. (2004) A role of OsGA20ox1, encoding an isoform of gibberellin 20‐oxidase, for regulation of plant stature in rice. Plant Mol. Biol. 55, 687–700. 15604710 10.1007/s11103-004-1692-y

[pbi13842-bib-0061] Oliva, R. , Ji, C. , Atienza‐Grande, G. , Huguet‐Tapia, J.C. , Perez‐Quintero, A. , Li, T. , Eom, J.S. *et al*. (2019) Broad‐spectrum resistance to bacterial blight in rice using genome editing. Nat. Biotechnol. 37, 1344–1350. 31659337 10.1038/s41587-019-0267-z PMC6831514

[pbi13842-bib-0062] Ordonio, R.L. , Ito, Y. , Hatakeyama, A. , Ohmae‐Shinohara, K. , Kasuga, S. , Tokunaga, T. , Mizuno, H. *et al*. (2014) Gibberellin deficiency pleiotropically induces culm bending in sorghum: an insight into sorghum semi‐dwarf breeding. Sci. Rep. 4, 1–10. 10.1038/srep05287 PMC4055941 24924234

[pbi13842-bib-0063] Østerberg, J.T. , Xiang, W. , Olsen, L.I. , Edenbrandt, A.K. , Vedel, S.E. , Christiansen, A. , Landes, X. *et al*. (2017) Accelerating the domestication of new crops: feasibility and approaches. Trends Plant Sci. 22, 373–384. 28262427 10.1016/j.tplants.2017.01.004

[pbi13842-bib-0064] Peng, J. , Richards, D.E. , Hartley, N.M. , Murphy, G.P. , Devos, K.M. , Flintham, J.E. , Beales, J. *et al*. (1999) ‘Green revolution’ genes encode mutant gibberellin response modulators. Nature, 400, 256–261. 10421366 10.1038/22307

[pbi13842-bib-0065] Peng, Y. , Hu, Y. , Qian, Q. and Ren, D. (2021) Progress and prospect of breeding utilization of green revolution gene SD1 in rice. Agriculture, 11, 611.

[pbi13842-bib-0066] Plaza‐Wüthrich, S. , Blösch, R. , Rindisbacher, A. , Cannarozzi, G. and Tadele, Z. (2016) Gibberellin deficiency confers both lodging and drought tolerance in small cereals. Front. Plant Sci. 7, 643. 27242844 10.3389/fpls.2016.00643 PMC4865506

[pbi13842-bib-0067] Qin, X. , Liu, J.H. , Zhao, W.S. , Chen, X.J. , Guo, Z.J. and Peng, Y.L. (2013) Gibberellin 20‐oxidase gene OsGA20ox3 regulates plant stature and disease development in rice. Mol. Plant Microbe Interact. 26, 227–239. 22992000 10.1094/MPMI-05-12-0138-R

[pbi13842-bib-0068] Quinby, J.R. and Karper, R.E. (1954) Inheritance of height in Sorghum 1. Agron. J. 46, 211–216.

[pbi13842-bib-0069] R Core Team (2013) R: A Language and Environment for Statistical Computing. Vienna: R Core Team.

[pbi13842-bib-0070] Sakamoto, T. , Miura, K. , Itoh, H. , Tatsumi, T. , Ueguchi‐Tanaka, M. , Ishiyama, K. , Kobayashi, M. *et al*. (2004a) An overview of gibberellin metabolism enzyme genes and their related mutants in rice. Plant Physiol. 134, 1642–1653. 15075394 10.1104/pp.103.033696 PMC419838

[pbi13842-bib-0071] Sakamoto, T. , Miyura, K. , Itoh, H. , Tatsumi, T. , Ueguchi‐Tanaka, M. , Ishiyama, K. , Kobayashi, M. *et al*. (2004b) Erratum: An overview of gibberellin metabolism enzyme genes and their related mutants in rice (Plant Physiology (2004) 134 (1642–1653)). Plant Physiol. 135, 1863. 10.1104/pp.103.033696 PMC419838 15075394

[pbi13842-bib-0072] Sasaki, A. , Ashikari, M. , Ueguchi‐Tanaka, M. , Itoh, H. , Nishimura, A. , Swapan, D. , Ishiyama, K. *et al*. (2002) A mutant gibberellin‐synthesis gene in rice: new insight into the rice variant that helped to avert famine over thirty years ago. Nature, 416, 701–702. 11961544 10.1038/416701a

[pbi13842-bib-0073] Shi, J. , Gao, H. , Wang, H. , Lafitte, H.R. , Archibald, R.L. , Yang, M. , Hakimi, S.M. *et al*. (2017) ARGOS 8 variants generated by CRISPR‐Cas9 improve maize grain yield under field drought stress conditions. Plant Biotechnol. J. 15, 207–216. 27442592 10.1111/pbi.12603 PMC5258859

[pbi13842-bib-0074] Shu, Q.‐Y. , Forster, B.P. , Nakagawa, H. and Nakagawa, H. (2012) Plant Mutation Breeding and Biotechnology. Wallingford, UK: Cabi.

[pbi13842-bib-0075] Spielmeyer, W. , Ellis, M.H. and Chandler, P.M. (2002) Semidwarf (sd‐1), “green revolution” rice, contains a defective gibberellin 20‐oxidase gene. Proc. Natl. Acad. Sci. USA, 99, 9043–9048. 12077303 10.1073/pnas.132266399 PMC124420

[pbi13842-bib-0076] Tefera, H. , Assefa, K. and Belay, G. (2003) Evaluation of interspecific recombinant inbred lines of *Eragrostis tef* x *E. pilosa* [Ethiopia]. J. Genet. Breed. 57, 21–30.

[pbi13842-bib-0077] Teklu, Y. and Tefera, H. (2005) Genetic improvement in grain yield potential and associated agronomic traits of tef (*Eragrostis tef*). Euphytica, 141, 247–254.

[pbi13842-bib-0078] Tomita, M. and Ishii, K. (2018) Genetic performance of the semidwarfing allele sd1 derived from a japonica rice cultivar and minimum requirements to detect its single‐nucleotide polymorphism by miseq whole‐genome sequencing. Biomed. Res. Int. 2018, 4241725. 29850513 10.1155/2018/4241725 PMC5903320

[pbi13842-bib-0079] Van Delden, S.H. , Vos, J. , Ennos, A.R. and Stomph, T.J. (2010) Analysing lodging of the panicle bearing cereal teff (*Eragrostis tef*). New Phytol. 186, 696–707. 20345637 10.1111/j.1469-8137.2010.03224.x

[pbi13842-bib-0080] VanBuren, R. , Man Wai, C. , Wang, X. , Pardo, J. , Yocca, A.E. , Wang, H. , Chaluvadi, S.R. *et al*. (2020) Exceptional subgenome stability and functional divergence in the allotetraploid Ethiopian cereal teff. Nat. Commun. 11, 1–11. 32060277 10.1038/s41467-020-14724-z PMC7021729

[pbi13842-bib-0081] Wang, N. , Arling, M. , Hoerster, G. , Ryan, L. , Wu, E. , Lowe, K. , Gordon‐Kamm, W. *et al*. (2020) An efficient gene excision system in Maize. Front. Plant Sci. 11, 1–13. 32983193 10.3389/fpls.2020.01298 PMC7492568

[pbi13842-bib-0082] Wang, Y. , Cheng, X. , Shan, Q. , Zhang, Y. , Liu, J. , Gao, C. and Qiu, J.L. (2014) Simultaneous editing of three homoeoalleles in hexaploid bread wheat confers heritable resistance to powdery mildew. Nat. Biotechnol. 32, 947–951. 25038773 10.1038/nbt.2969

[pbi13842-bib-0083] Waterhouse, A.M. , Procter, J.B. , Martin, D.M.A. , Clamp, M. and Barton, G.J. (2009) Jalview Version 2—a multiple sequence alignment editor and analysis workbench. Bioinformatics, 25, 1189–1191. 19151095 10.1093/bioinformatics/btp033 PMC2672624

[pbi13842-bib-0084] Yamaguchi, M. , Fujimoto, H. , Hirano, K. , Araki‐Nakamura, S. , Ohmae‐Shinohara, K. , Fujii, A. , Tsunashima, M. *et al*. (2016) Sorghum Dw1, an agronomically important gene for lodging resistance, encodes a novel protein involved in cell proliferation. Sci. Rep. 6, 1–11. 27329702 10.1038/srep28366 PMC4916599

[pbi13842-bib-0085] Yamaguchi, S. (2008) Gibberellin metabolism and its regulation. Annu. Rev. Plant Biol. 59, 225–251. 18173378 10.1146/annurev.arplant.59.032607.092804

[pbi13842-bib-0086] Zhu, H. , Li, C. and Gao, C. (2020) Applications of CRISPR–Cas in agriculture and plant biotechnology. Nat. Rev. Mol. Cell Biol. 21, 661–677. 32973356 10.1038/s41580-020-00288-9

[pbi13842-bib-0087] Zhu, Q. , Smith, S.M. , Ayele, M. , Yang, L. , Jogi, A. , Chaluvadi, S.R. and Bennetzen, J.L. (2012) High‐throughput discovery of mutations in tef semi‐dwarfing genes by next‐generation sequencing analysis. Genetics, 192, 819–829. 22904035 10.1534/genetics.112.144436 PMC3522160

[pbi13842-bib-0088] Zsögön, A. , Čermák, T. , Naves, E.R. , Notini, M.M. , Edel, K.H. , Weinl, S. , Freschi, L. *et al*. (2018) De novo domestication of wild tomato using genome editing. Nat. Biotechnol. 36, 1211–1216. 10.1038/nbt.4272 30272678

