# Supplementary material for: CRISPR/Cas9‐mediated tetra‐allelic mutation of the ‘Green Revolution’ SEMIDWARF‐1 (SD‐1) gene confers lodging resistance in tef (Eragrostis tef)
Source: Plant Biotechnol J. 2022 Jun 10;20(9):1716–29. doi: 10.1111/pbi.13842 (PMC9398311; doi:10.1111/pbi.13842)
Supplement: Supplementary file 1 — Figure S1 Plasmid maps of p8660 used to generate transgenic and gene‐edited tef lines. Figure S2 Plasmid map of p8702 used to generate transgenic and gene‐edited tef lines. Figure S3 GA3 treatment effect on stem elongation in the greenhouse‐grown (a, b) and in vitro grown (c, d) plants of SD‐1 knockout lines (8660‐6, 8660‐12) and wild‐type control. Figure S4 Lodging in sd‐1 tef line and wild‐type Magna control. [file PBI-20-1716-s002.pdf]

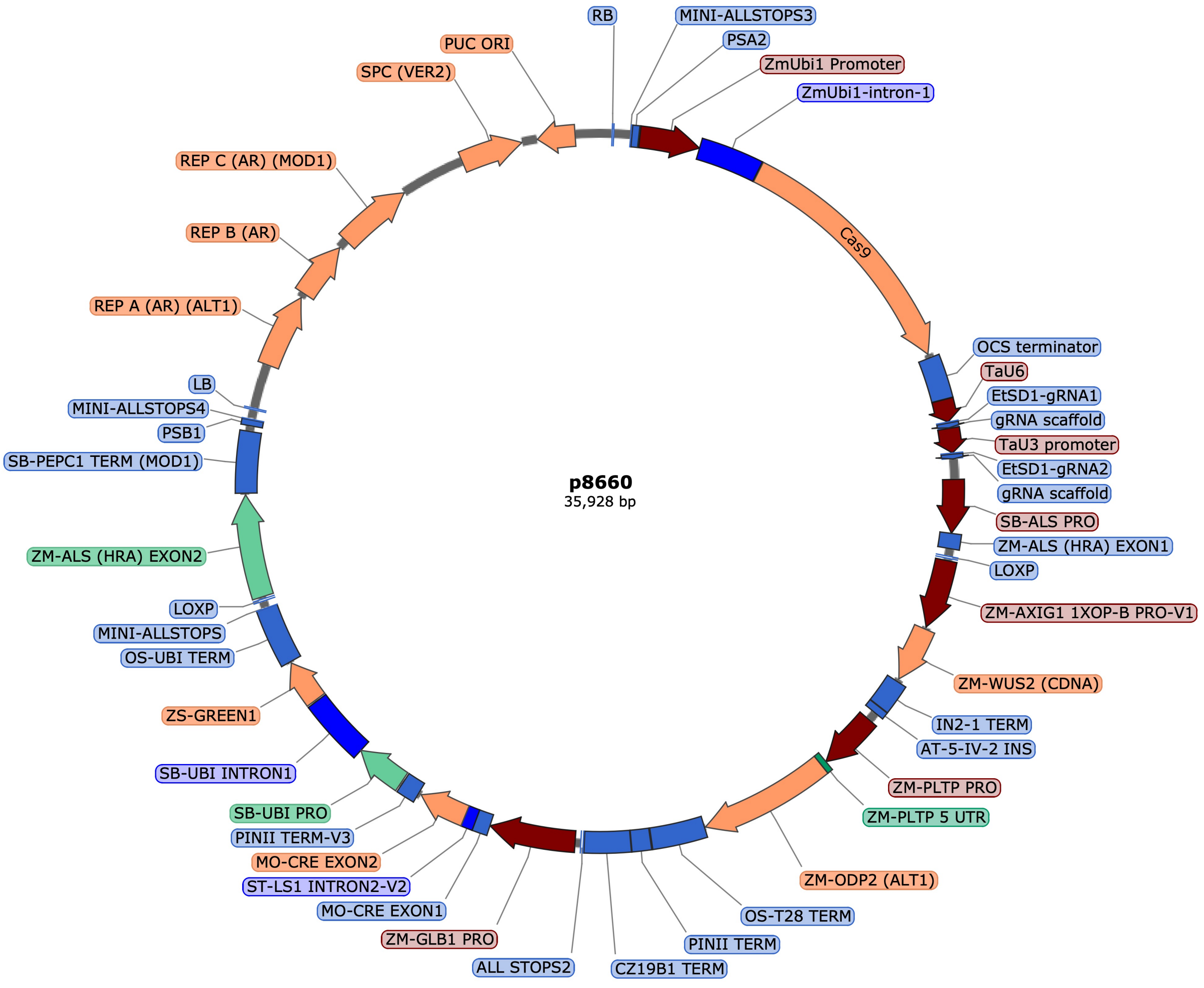

Supplementary Figure S1. Plasmid map of p8660 used to generate transgenic and gene edited tef lines.

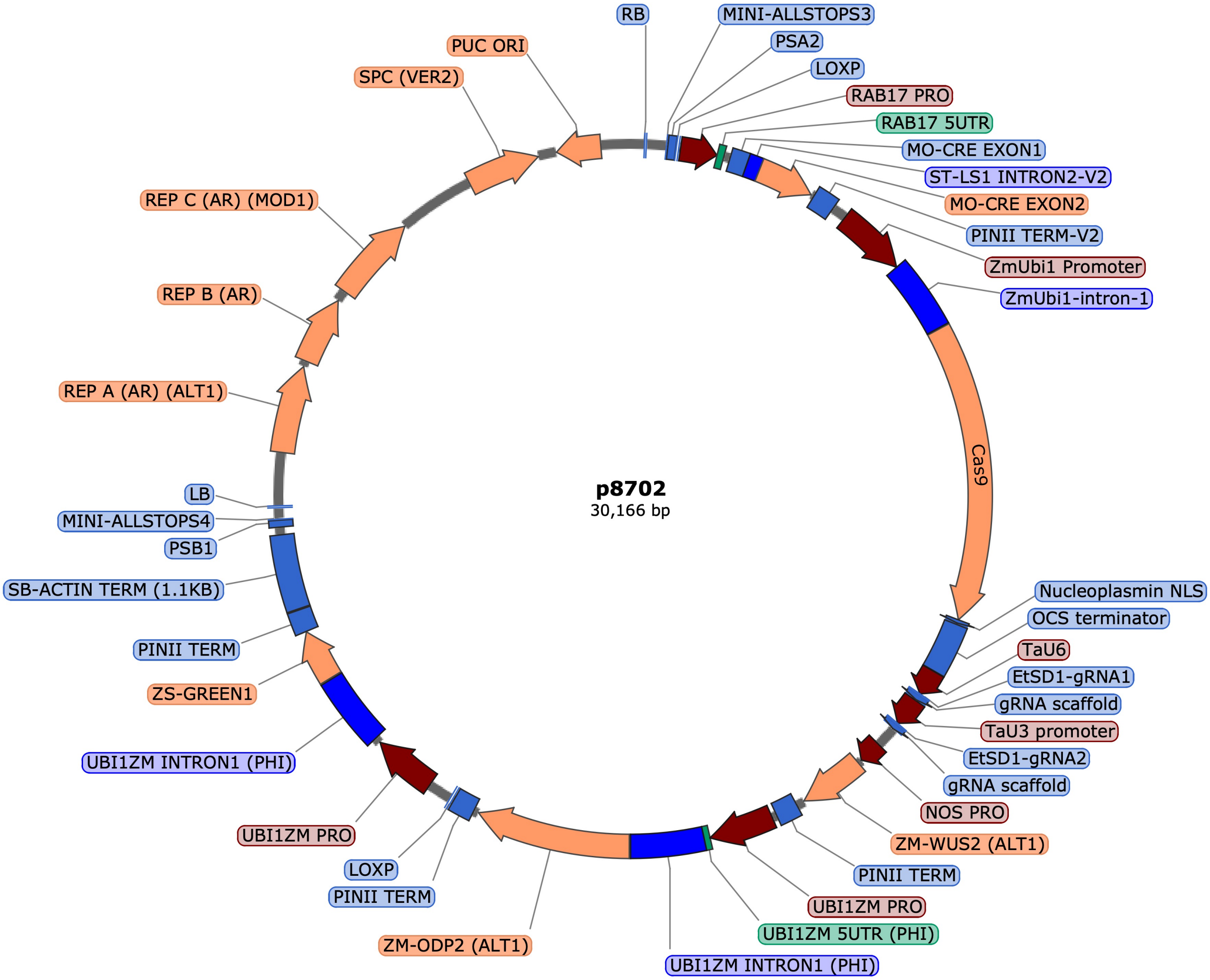

Supplementary Figure S2. Plasmid map of p8702 used to generate transgenic and gene edited tef lines.

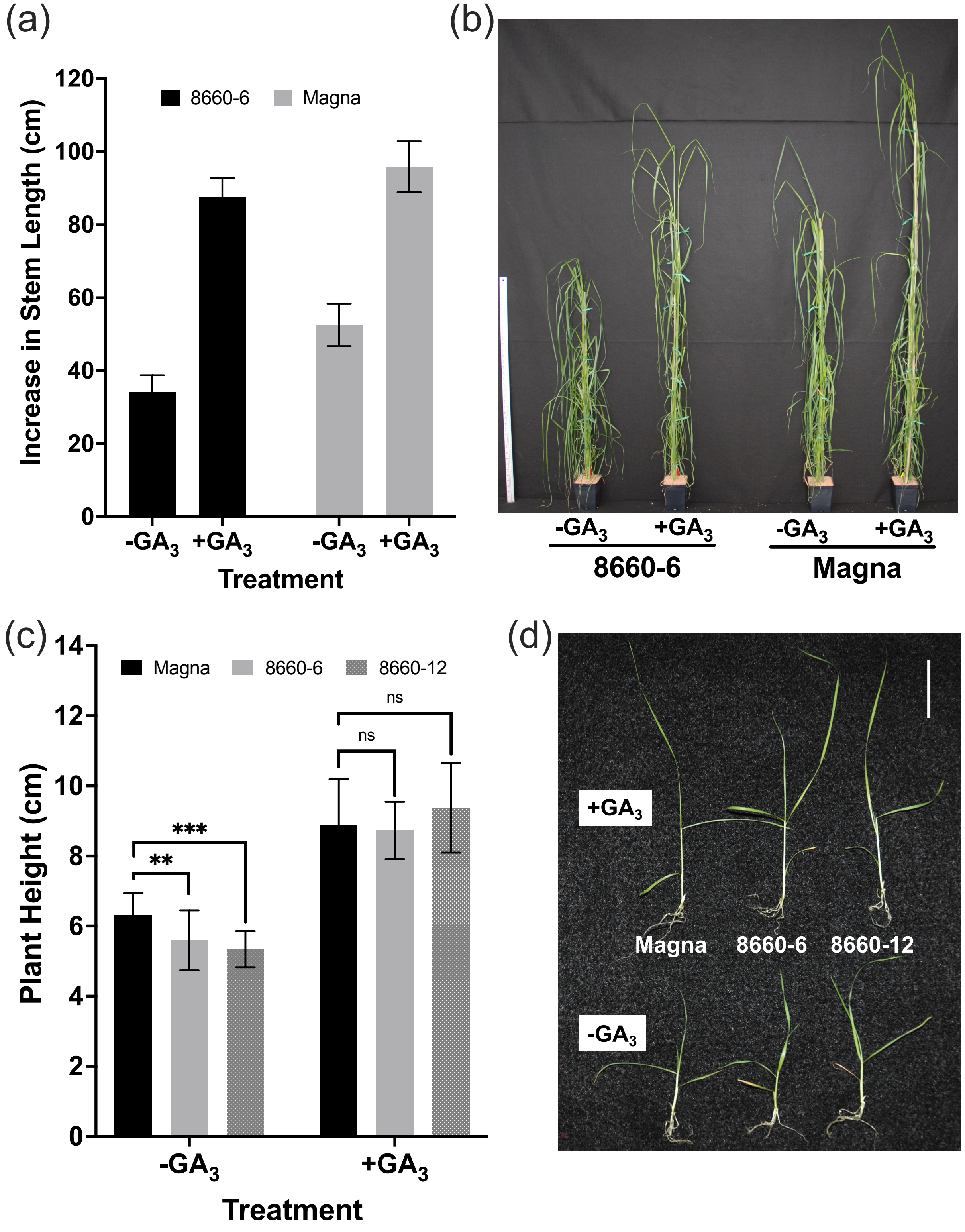

Supplementary Figure S3. GA_3 treatment effect on stem elongation in the greenhouse grown (a, b) and in vitro grown (c, d) plants of *SD-1* knockout lines (8660-6, 8660-12) and wild-type control. Effect of GA_3 treatment in the greenhouse (a, b) was measured as an increase in plant height after 11 days treatment period. Bars show means ± SD, n = 9 (a) and n = 20 (c). **, and ***, stand for significant differences, respectively, at $p \leq 0.01$, and $p \leq 0.001$. ns, stands for non-significant differences at $p<0.05$. Student's *t*-test was used for comparison. Scale bar in d = 2 cm.

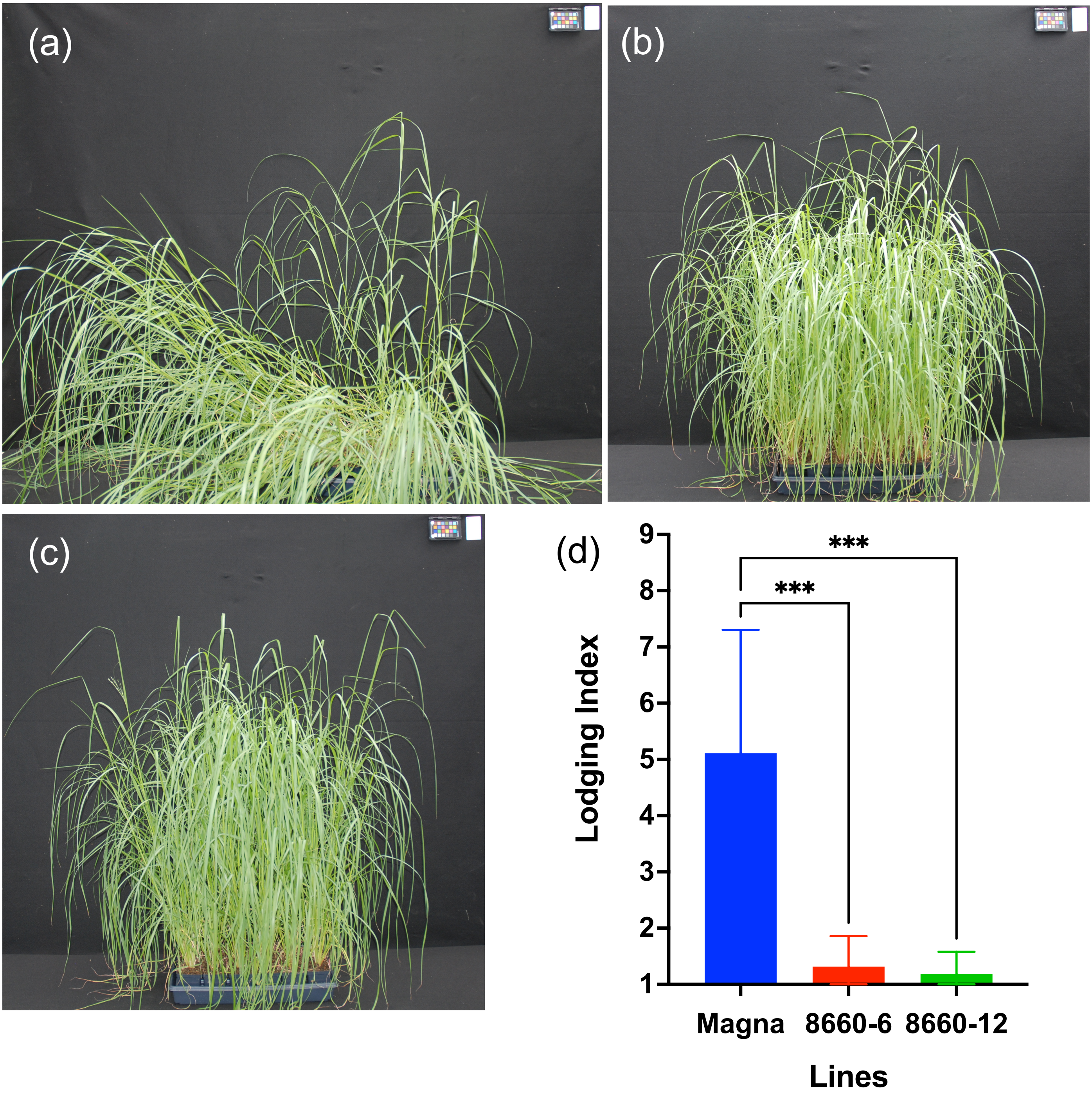

Supplementary Figure S4. Lodging in *sd-1* tef line and wild-type Magna control. Lodging (falling or bending of tef plant stems from upright growth in Magna (a) and T_2 *sd-1* line 8660-6 (b) and 8660-12 (c) at 8 weeks after planting (heading stage). Estimate of lodging indices as per USDA-GRINs descriptors for tef (d). ***, stands for significant differences at *p<0.001*.
