# Supplementary material for: CRISPR/Cas9‐mediated tetra‐allelic mutation of the ‘Green Revolution’ SEMIDWARF‐1 (SD‐1) gene confers lodging resistance in tef (Eragrostis tef)
Source: Plant Biotechnol J. 2022 Jun 10;20(9):1716–29. doi: 10.1111/pbi.13842 (PMC9398311; doi:10.1111/pbi.13842)
Supplement: Supplementary file 2 — Table S1 Nucleotide sequence identity among rice and tef GA 20‐oxidases Table S2 Amino acid sequence identity among rice and tef GA 20‐oxidases Table S3 Transformation efficiency in two tef cultivars using young leaf explants Table S4 SD‐1 mutation detected at target site 1 and target site 2 in both subgenome‐A and subgenome‐B Table S5 Sequences of guide RNAs (gRNAs) oligos targeting tef SD‐1 and sequencing primers for confirmation of cloned gRNAs and Cas9 Table S6 Primers used to generate target site amplicons for Next‐Generation Sequencing (NGS) of SD‐1 gene mutation Table S7 Quantitative PCR (qPCR) primers used to detect T‐DNA in T1/T2 plants [file PBI-20-1716-s001.pdf]

Supplementary Table S1. Nucleotide sequence identity among rice and tef GA 20-oxidases.

	Et_5B_044286	Et_5A_041632	Et_3A_025601	Et_3B_029946	Et_4A_035650	Et_4B_039790	Et_9A_061857	Os01g66100	Os05g34854	Os03g63970	Os07g07420
**Et_5B_044286**											
**Et_5A_041632**	95.40										
**Et_3A_025601**	69.40	68.00									
**Et_3B_029946**	69.40	68.76	94.20								
**Et_4A_035650**	70.70	71.22	68.46	67.78							
**Et_4B_039790**	71.00	71.76	69.35	68.09	90.79						
**Et_9A_061857**	65.20	64.99	75.65	75.53	62.93	61.72					
**Os01g66100**	67.10	65.95	83.53	83.55	67.48	67.06	76.30				
**Os05g34854**	67.20	65.44	76.73	76.64	65.88	64.31	76.72	78.48			
**Os03g63970**	70.80	70.40	67.71	67.51	76.70	76.79	61.46	65.47	64.72		
**Os07g07420**	86.90	85.94	70.13	70.17	70.27	69.85	65.17	67.21	68.90	70.55	

Supplementary Table S2. Amino acid sequence identity among rice and tef GA 20-oxidases.

	Et_5B_044286	Et_5A_041632	Et_3A_025601	Et_3B_029946	Et_4A_035650	Et_4B_039790	Et_9A_061857	Os01g66100	Os05g34854	Os03g63970	Os07g07420
**Et_5B_044286**											
**Et_5A_041632**	88.20										
**Et_3A_025601**	53.61	51.15									
**Et_3B_029946**	52.23	50.16	92.27								
**Et_4A_035650**	58.19	56.15	56.23	54.31							
**Et_4B_039790**	58.15	55.49	53.98	52.38	88.47						
**Et_9A_061857**	48.80	46.49	69.30	68.21	49.42	49.14					
**Os01g66100**	50.29	48.55	84.43	81.67	52.38	50.97	66.02				
**Os05g34854**	50.57	48.90	63.98	62.63	48.92	49.14	66.58	65.01			
**Os03g63970**	57.34	54.72	55.33	53.35	71.31	69.23	48.55	51.13	46.43		
**Os07g07420**	79.94	77.12	54.79	53.10	58.19	57.87	49.55	51.87	50.70	57.75	

Supplementary Table S3. Transformation efficiency in two tef cultivars using young leaf explants

**Construct**	**Cultivar**	**Number of Transformations**[1]	**Number of Explant Infected**	**Number of Rooted T_0 Plants (% Efficiency)**[2]
p8660	Magna	5	152	31 (N/A)
p8702	ADA	7	525	41 (7.81)
p8702	Magna	5	248	41 (16.53)

1 Number of transformation experiments run on different dates

2 Percent efficiency is number of rooted T_0 events recovered per 100 explants. Percent efficiency for the construct 8660 is not presented as multiple rooted plants (some clonal have been recovered from a few callus lines).

Supplementary Table S4. *SD-1* mutation detected at target site 1 and target site 2 in both subgenome-A and subgenome-B. Data of mutation detected presented as percentage of the total reads obtained by next generation sequencing of the target site amplicons.

	Sub-Genome A gRNA1				Genome A gRNA2				Genome B gRNA1				Genome B gRNA2			
SampleName	Target Site Sequence	AlleleChange	#AlleleReads	%AlleleRead	Target Site Sequence	AlleleChange	#AlleleReads	%AlleleRead	Target Site Sequence	AlleleChange	#AlleleReads	%AlleleRead	Target Site Sequence	AlleleChange	#AlleleReads	%AlleleRead
Magna_8660-1	GAGCCAATGG	'+A	97497	100	AGGACAGCCG	'+T	38439	66.9133	GAGCCAATGG	'-C	44041	51.3502	AGGACAGCCG	'+T	18266	47.4947
			97497	Total Muta.		Wild Type	13773	23.9756		'+A	41725	48.6498		Wild Type	14570	37.8845
			100.0%	% NHEJ		'+A	5234	9.1112			85766	Total Muta.		'+A	5623	14.6208
							43673	Total Muta.			100.0%	% NHEJ			23889	Total Muta.
							76.0245%	% NHEJ							62.1155%	% NHEJ
Magna_8660-2	GAGCCAATGG	'+A	36151	53.8378	AGGACAGCCG	'+T	20889	41.3324	GAGCCAATGG	'+A	19293	39.1331	AGGACAGCCG	'+A	23545	67.5435
		'-GC	30997	46.1622		'+A	20746	41.0495		'-AGCC	17699	35.8999		'+T	6759	19.3895
			67148	Total Muta.		Wild Type	8904	17.6181		'-C	12309	24.967		Wild Type	4555	13.0669
			100.0%	% NHEJ			41635	Total Muta.			49301	Total Muta.			30304	Total Muta.
							82.3819%	% NHEJ			100.0%	% NHEJ			86.933%	% NHEJ
Magna_8660-3	GAGCCAATGG	'+A	71481	100	AGGACAGCCG	'+T	31396	66.3104	GAGCCAATGG	'-C	32651	52.7701	AGGACAGCCG	'+T	18281	59.1503
			71481	Total Muta.		Wild Type	8839	18.6686		'+A	29223	47.2299		'Indel before Target	12625	40.8497
			100.0%	% NHEJ		'+A	7112	15.021			61874	Total Muta.			30906	Total Muta.
							38508	Total Muta.			100.0%	% NHEJ			100.0%	% NHEJ
							81.3314%	% NHEJ								
Magna_8660-4	GAGCCAATGG	'+A	30844	54.1208	AGGACAGCCG	'+T	22499	41.6101	GAGCCAATGG	Wild Type	15457	36.2203	AGGACAGCCG	'+A	19748	62.4285
		'-GC	26147	45.8792		'+A	18348	33.9332		'+A	14553	34.1019		Wild Type	6910	21.8443
			56991	Total Muta.		Wild Type	13224	24.4567		'-C	12665	29.6778		'+T	4975	15.7272
			100.0%	% NHEJ			40847	Total Muta.			27218	Total Muta.			24723	Total Muta.
							75.5433%	% NHEJ			63.7797%	% NHEJ			78.1557%	% NHEJ
Magna_8660-5	GAGCCAATGG	'+A	83778	100	AGGACAGCCG	'+G	26559	45.4762	GAGCCAATGG	'-C	35737	50.1671	AGGACAGCCG	Wild Type	28934	82.5059
			83778	Total Muta.		'+T	26485	45.3495		'+A	35499	49.8329		'+T	3891	11.0953
			100.0%	% NHEJ		Wild Type	5358	9.1743			71236	Total Muta.		'+G	2244	6.3988
							53044	Total Muta.			100.0%	% NHEJ			6135	Total Muta.
							90.8257%	% NHEJ							17.4941%	% NHEJ
Magna_8660-6	GAGCCAATGG	'+A	40394	54.7745	AGGACAGCCG	'+T	35366	52.8624	GAGCCAATGG	'Indel before Target	22967	71.1053	AGGACAGCCG	'+A	19196	49.0557
		'-GC	33352	45.2255		Wild Type	15915	23.7885		'+A	9333	28.8947		Wild Type	15797	40.3695
			73746	Total Muta.		'+A	15621	23.3491			32300	Total Muta.		'+T	4138	10.5747
			100.0%	% NHEJ			50987	Total Muta.			100.0%	% NHEJ			23334	Total Muta.
							76.2115%	% NHEJ							59.6304%	% NHEJ
Magna_8660-7	GAGCCAATGG	'+A	73541	83.9021	AGGACAGCCG	'+T	46587	100	GAGCCAATGG	'+A	44843	50.1588	AGGACAGCCG	'+T	14603	50.0926
		'-C	14110	16.0979			46587	Total Muta.		'-C	44559	49.8412		'Indel before Target	9714	33.3219
			87651	Total Muta.			100.0%	% NHEJ			89402	Total Muta.		'+A	4835	16.5855
			100.0%	% NHEJ							100.0%	% NHEJ			29152	Total Muta.
															100.0%	% NHEJ
Magna_8660-8	GAGCCAATGG	'-GC	44749	68.1993	AGGACAGCCG	'+A	45476	80.5498	GAGCCAATGG	'-C	19960	43.6637	AGGACAGCCG	'+A	25127	79.063
		'+A	16870	25.7106		'+T	10981	19.4502		'+A	14430	31.5665		'+T	6654	20.937
		'-GC; A to C	3996	6.0901			56457	Total Muta.		'-AGCC	7447	16.2908			31781	Total Muta.
			65615	Total Muta.			100.0%	% NHEJ		'-GCC	3876	8.479			100.0%	% NHEJ
			100.0%	% NHEJ							45713	Total Muta.				
											100.0%	% NHEJ				
Magna_8660-9	GAGCCAATGG	'+A	33490	54.7374	AGGACAGCCG	Wild Type	17288	41.5198	GAGCCAATGG	'-C	30933	80.31	AGGACAGCCG	'+A	35628	94.1917
		'-GC	27693	45.2626		'+A	16765	40.2637		'+A	7584	19.69		Wild Type	2197	5.8083
			61183	Total Muta.		'+T	7585	18.2165			38517	Total Muta.			35628	Total Muta.
			100.0%	% NHEJ			24350	Total Muta.			100.0%	% NHEJ			94.1917%	% NHEJ
							58.4802%	% NHEJ								
Magna_8660-10	GAGCCAATGG	'+A	67885	100	AGGACAGCCG	'+T	45954	83.4329	GAGCCAATGG	'-C	43648	53.4633	AGGACAGCCG	'+T	12618	37.9671
			67885	Total Muta.		Wild Type	5627	10.2162		'+A	37993	46.5367		'+A	9935	29.8941
			100.0%	% NHEJ		'+A	3498	6.3509			81641	Total Muta.		Wild Type	7966	23.9694
							49452	Total Muta.			100.0%	% NHEJ		'-GA	2715	8.1693
							89.7838%	% NHEJ							25268	Total Muta.
															76.0305%	% NHEJ
Magna_8660-12	GAGCCAATGG	'+A	85013	100	AGGACAGCCG	'+A	24160	53.6186	GAGCCAATGG	'-C	48989	54.2802	AGGACAGCCG	'+A	34398	77.6584
			85013	Total Muta.		'+T	20899	46.3814		'+A	41263	45.7198		'+T	9896	22.3416
			100.0%	% NHEJ			45059	Total Muta.			90252	Total Muta.			44294	Total Muta.
							100.0%	% NHEJ			100.0%	% NHEJ			100.0%	% NHEJ
Magna_8660-13	GAGCCAATGG	'+A	57532	82.8335	AGGACAGCCG	'+T	41781	100	GAGCCAATGG	'+A	53379	100	AGGACAGCCG	'Indel before Target	8150	38.8817
		'-C	11923	17.1665			41781	Total Muta.			53379	Total Muta.		'+T	6958	33.195
			69455	Total Muta.			100.0%	% NHEJ			100.0%	% NHEJ		'+A	4387	20.9293
			100.0%	% NHEJ										'+C	1466	6.9939
															20961	Total Muta.
															99.9999%	% NHEJ

Magna_8660-14	GAGCCAATGG	'-GC	24878	50.991	AGGACAGCCG	'+A	20408	61.6184	GAGCCAATGG	'+A	66488	100	AGGACAGCCG	'+A	13986	62.8274
		'+A	23911	49.009		'+T	12712	38.3816			66488	Total Muta.		'Indel before Target	8275	37.1726
			48789	Total Muta.			33120	Total Muta.			100.0%	% NHEJ			22261	Total Muta.
			100.0%	% NHEJ			100.0%	% NHEJ							100.0%	% NHEJ
Magna_8660-15	GAGCCAATGG	'+A	36248	53.1379	AGGACAGCCG	'+A	37854	69.4684	GAGCCAATGG	'+A	59758	100	AGGACAGCCG	'+A	40270	92.5194
		'-GC	31967	46.8621		'+T	12913	23.6975			59758	Total Muta.		'+T	3256	7.4806
			68215	Total Muta.		Wild Type	3724	6.8342			100.0%	% NHEJ			43526	Total Muta.
			100.0%	% NHEJ			50767	Total Muta.							100.0%	% NHEJ
							93.1659%	% NHEJ								
Magna_8660-16	GAGCCAATGG	'+A	73158	100	AGGACAGCCG	'+T	30659	76.7992	GAGCCAATGG	'-C	27332	57.1178	AGGACAGCCG	'+T	20799	53.6998
			73158	Total Muta.		'+A	5883	14.7366		'+A	20520	42.8822		'+A	17933	46.3002
			100.0%	% NHEJ		'Indel before Target	3379	8.4642			47852	Total Muta.			38732	Total Muta.
							39921	Total Muta.			100.0%	% NHEJ			100.0%	% NHEJ
							100.0%	% NHEJ								
Magna_8660-17	GAGCCAATGG	'+A	32814	56.7589	AGGACAGCCG	'+T	35933	63.2145	GAGCCAATGG	'-C	34848	56.1051	AGGACAGCCG	'+A	18320	81.8296
		'+AA	24999	43.2411		'+A	13435	23.6353		'+A	27264	43.8949		'+T	4068	18.1704
			57813	Total Muta.		'+G	7475	13.1503			62112	Total Muta.			22388	Total Muta.
			100.0%	% NHEJ			56843	Total Muta.			100.0%	% NHEJ			100.0%	% NHEJ
							100.0001%	% NHEJ								
Magna_8660-18	GAGCCAATGG	'+A	44089	100	AGGACAGCCG	'+T	51753	100	GAGCCAATGG	'+A	15246	53.993	AGGACAGCCG	'Indel before Target	6799	41.2111
			44089	Total Muta.			51753	Total Muta.		'Indel before Target	7502	26.568		'+A	5741	34.7982
			100.0%	% NHEJ			100.0%	% NHEJ		'-GCC	5489	19.439		'+T	3958	23.9908
											28237	Total Muta.			16498	Total Muta.
											100.0%	% NHEJ			100.0001%	% NHEJ
Magna_8660-19	GAGCCAATGG	'+A	77066	100	AGGACAGCCG	'+T	51258	94.1879	GAGCCAATGG	'-A	43401	51.528	AGGACAGCCG	'+A	13474	53.9975
			77066	Total Muta.		'+A	3163	5.8121		'-C	40827	48.472		'Indel before Target	8374	33.5591
			100.0%	% NHEJ			54421	Total Muta.			84228	Total Muta.		'+T	3105	12.4434
							100.0%	% NHEJ			100.0%	% NHEJ			24953	Total Muta.
															100.0%	% NHEJ
Ada_8702-3	GAGCCAATGG	Wild Type	31293	50.4596	AGGACAGCCG	'+T	33364	45.1389	GAGCCAATGG	Wild Type	43274	51.0541	AGGACAGCCG	'+A	35218	88.5163
		'+A	30723	49.5404		Wild Type	32363	43.7847		'-C	41487	48.9459		'+T	2331	5.8587
			30723	Total Muta.		'+A	8187	11.0764			41487	Total Muta.		Wild Type	2238	5.625
			49.5404%	% NHEJ			41551	Total Muta.			48.9459%	% NHEJ			37549	Total Muta.
							56.2153%	% NHEJ							94.375%	% NHEJ
Ada_8702-4	GAGCCAATGG	'Indel before Targ	31379	57.9986	AGGACAGCCG	'+A	43144	54.4906	GAGCCAATGG	'+A	22114	50.9774	AGGACAGCCG	'+A	35449	93.1422
		'-GC	22724	42.0014		'+T	36033	45.5094		'-C	21266	49.0226		'+T	2610	6.8578
			54103	Total Muta.			79177	Total Muta.			43380	Total Muta.			38059	Total Muta.
			100.0%	% NHEJ			100.0%	% NHEJ			100.0%	% NHEJ			100.0%	% NHEJ
Ada_8702-5	GAGCCAATGG	'+A	36806	51.2376	AGGACAGCCG	'+A	35504	46.0702	GAGCCAATGG	'-C	56400	50.2338	AGGACAGCCG	Wild Type	35558	77.2027
		Wild Type	30675	42.7026		'+T	34774	45.1229		'+A	55875	49.7662		'+A	7368	15.9972
		'-AGCC	4353	6.0598		Wild Type	6787	8.8069			112275	Total Muta.		'+T	3132	6.8001
			41159	Total Muta.			70278	Total Muta.			100.0%	% NHEJ			10500	Total Muta.
			57.2974%	% NHEJ			91.1931%	% NHEJ							22.7973%	% NHEJ
Ada_8702-6	GAGCCAATGG	'+C	30804	53.3172	AGGACAGCCG	'+T	40052	52.7987	GAGCCAATGG	'+A	68032	82.7348	AGGACAGCCG	'+T	24039	50.2876
		'-AGCC	15461	26.7607		Wild Type	35806	47.2013		Wild Type	14197	17.2652		'+A	20645	43.1877
		Wild Type	11510	19.9221			40052	Total Muta.			68032	Total Muta.		Wild Type	3119	6.5247
			46265	Total Muta.			52.7987%	% NHEJ			82.7348%	% NHEJ			44684	Total Muta.
			80.0779%	% NHEJ											93.4753%	% NHEJ
Ada_8702-7	GAGCCAATGG	'+A	32317	54.4222	AGGACAGCCG	'+T	33115	82.3081	GAGCCAATGG	'-C	49124	50.2743	AGGACAGCCG	Wild Type	22631	56.4618
		'-GCC	19923	33.5506		'+A	4844	12.0399		'+A	48588	49.7257		'-A	12517	31.2285
		'-AGCC	7142	12.0272		Wild Type	2274	5.6521			97712	Total Muta.		'+T	4934	12.3098
			59382	Total Muta.			37959	Total Muta.			100.0%	% NHEJ			17451	Total Muta.
			100.0%	% NHEJ			94.348%	% NHEJ							43.5383%	% NHEJ

Note: In Ada background, 8702-1, 8702-2 and 8702-4 are likely clones, while in Magna cultivar background 8660-1 and 8660-11 and 8660-7 and 8660-20 are also likely clones.

Supplementary Table S5. Sequences of guide RNAs (gRNAs) oligos targeting tef *SD-1* and sequencing primers for confirmation of cloned gRNAs and Cas9.

Primer name	primer	Sequence
gRNA1-F	forward	acttGGCCCGGACTTCGAGCCAATg
gRNA1-R	reverse	aaaacATTGGCTCGAAGTCCGGGCC
gRNA2-F	forward	gaagGCGACTTCTTCGAGGACAGCg
gRNA2-R	reverse	aaaacGCTGTCCTCGAAGAAGTCGC
gRNA1-seq	sequencing	GACCAAGCCCGTTATTCTGACA
gRNA2-seq	sequencing	GGCAGGGAGAGTTTTAACATTGAC
Cas9-seq	sequencing	TCGTGTACGGGGACTACAAGGTTT
Cas9-term.seq	sequencing	CCGGTCAAACCTAAAAGACTGATTAC

Note: sequences in lower case are added to make oligos commutable with intermediate cloning vectors.

Supplementary Table S6. Primers used to generate target site amplicons for Next-Generation Sequencing (NGS) of *SD-1* gene mutation.

Target	primer	Sequence
A-gRNA1F	forward	atcgggaagctgaagCTGTCCTTCCGCTTCCAC
A-gRNA1R	reverse	atccgacggtagtgtCCAGTCAACGTGGTTGGT
A-gRNA2F	forward	atcgggaagctgaagATCATGGAGCTGCTGGAG
A-gRNA2R	reverse	atccgacggtagtgtGGTAGTAGTTGCACCGCA
B-gRNA1F	forward	atcgggaagctgaagGGAGACGCTGTCCTTCGGCT
B-gRNA1R	reverse	atccgacggtagtgtTATCCACAGCAAGCCCGCC
B-gRNA2F	forward	atcgggaagctgaagGTCCCTGACGATCATGGA
B-gRNA2R	reverse	atccgacggtagtgtGGTAGTAGTTGCACCGCA

Note: sequences in lower case are NGS adaptor sequences, and they are the primers used in secondary PCR.

Supplementary Table S7. Quantitative PCR (qPCR) primers used to detect T-DNA in T_1/T_2 plants

primer name	primers	Sequence
PSA-F	forward	CATGAAGCGCTCACGGTTACTAT
PSA-R	reverse	TCGTACGCTACTGCCACCAA
PSA-probe	FAM MGB	ACGGTTAGCTTCACGACT
ODP-F	forward	CGGCGATGTCTGCTTCAA
ODP-R	reverse	AAGCTCTGATCCCCTCATGCT
ODP-probe	FAM MGB	ATCCCCCAAGATTG
GREEN-F	forward	GACGGCCACAAGTTCGTGAT
GREEN-R	reverse	AGGTTGATGGCCTGCTTGC
GREEN-probe	FAM MGB	TAGCCGATGCCCTCG
PSB-F	forward	TGATTCCGATGACTTCGTAGGTT
PSB-R	reverse	GCTAATCGTAAGTGACGCTTGGA
PSB-probe	FAM MGB	CTAGCTCAAGCCGCTC
TEF PDS-F	forward	GGCTGGTCTATCAACAGCAAAGTAT
TEF PDS-R	reverse	ATGTAACCATCAGACCTTTCCACC
TEF PDS-probe	VIC MGB	TGAGGCAAGGGATGTT
